# Social disconnectedness, subsequent medical conditions, and, the role of pre-existing mental disorders: a population-based cohort study

**DOI:** 10.1017/S2045796024000829

**Published:** 2024-12-23

**Authors:** L. M. Laustsen, M. Lasgaard, N. C. Momen, D. Chen, J. L. Gradus, M. S. Grønkjær, M. M. Jensen, O. Plana-Ripoll

**Affiliations:** 1 Department of Clinical Epidemiology, Aarhus University and Aarhus University Hospital, Aarhus, Denmark; 2 DEFACTUM – Public Health Research, Central Denmark Region, Aarhus, Denmark; 3 Department of Psychology, University of Southern Denmark, Odense, Denmark; 4 Department of Epidemiology, Boston University School of Public Health, Boston, MA, USA; 5 Department of Psychiatry, Boston University School of Medicine, Boston, MA, USA; 6 Center for Clinical Research and Prevention, Copenhagen University Hospital – Bispebjerg and Frederiksberg, Copenhagen, Denmark; 7 National Centre for Register-based Research, Aarhus University, Aarhus, Denmark

**Keywords:** chronic conditions, epidemiology, mental health, risk factors, social network

## Abstract

**Aims:**

Individuals with diminished social connections are at higher risk of mental disorders, dementia, circulatory conditions and musculoskeletal conditions. However, evidence is limited by a disease-specific focus and no systematic examination of sex differences or the role of pre-existing mental disorders.

**Methods:**

We conducted a cohort study using data on social disconnectedness (loneliness, social isolation, low social support and a composite measure) from the 2013 and 2017 Danish National Health Survey linked with register data on 11 broad categories of medical conditions through 2021. Poisson regression was applied to estimate incidence rate ratios (IRRs), incidence rate differences (IRDs), and explore sex differences and interaction with pre-existing mental disorders.

**Results:**

Among 162,497 survey participants, 7.6%, 3.5% and 14.8% were classified as lonely, socially isolated and with low social support, respectively. Individuals who were lonely and with low social support had a higher incidence rate in all 11 categories of medical conditions (interquartile range [IQR] of IRRs, respectively 1.26–1.49 and 1.10–1.14), whereas this was the case in nine categories among individuals who were socially isolated (IQR of IRRs, 1.01–1.31). Applying the composite measure, the highest IRR was 2.63 for a mental disorder (95% confidence interval [CI], 2.38–2.91), corresponding to an IRD of 54 (95% CI, 47–61) cases per 10,000 person-years. We found sex and age differences in some relative and absolute estimates, but no substantial deviations from additive interaction with pre-existing mental disorders.

**Conclusions:**

This study advances our knowledge of the risk of medical conditions faced by individuals who are socially disconnected. In addition to the existing evidence, we found higher incidence rates for a broad range of medical condition categories. Contrary to previous evidence, our findings suggest that loneliness is a stronger determinant for subsequent medical conditions than social isolation and low social support.

A preregistered analysis plan and statistical code are available at Open Science Framework (https://osf.io/pycrq).

## Introduction

Individuals with diminished social connections are at substantially higher risk of developing several medical conditions (Leigh-Hunt *et al.*, 2017, Liang *et al.*, 2024) and premature death due to these medical conditions (Wang *et al.*, 2023a). Systematic reviews have shown a higher risk of depression (Mann *et al.*, 2022), dementia (Wang *et al.*, 2023b), coronary heart disease and stroke (Valtorta *et al.*, 2016) and sarcopenia (Yang *et al.*, 2023) (for a full overview of previous reviews, see Table S1). The current evidence is, however, limited with regard to other medical conditions and potential constraints related to sample size, as only two (Mann *et al.*, 2022; Valtorta *et al.*, 2016) of the identified systematic reviews have an accumulated study population of more than 80,000 (see Table S1). For medical conditions with a low incidence, the sample size is important to identify associations with moderate and low strength. Thus, there is a need to investigate the association between social disconnectedness and a wide range of medical conditions in a large representative sample. Additionally, prior studies are mainly characterized by a narrower focus on one outcome, which complicates comparisons between different medical conditions. Furthermore, potential sex and age differences have not systematically been examined despite suggested sex (Wang *et al.*, 2023a) and age (Mann *et al.*, 2022) differences for loneliness. Given that mental disorders are highly correlated with both social disconnectedness (Pearce *et al.*, 2023) and medical conditions (Momen *et al.*, 2020), it is also relevant to explore the role of pre-existing mental disorders in these associations. In our recent study (Laustsen *et al.*, 2024), we found substantial interaction between social disconnectedness and mental disorders on mortality among men, but only three prior studies have explored this interplay with regard to subsequent medical conditions (Enga *et al.*, 2012; Fang *et al.*, 2019; Guo *et al.*, 2023).

The aim of this study was to provide a comprehensive overview of relative and absolute differences in the incidence rates of 11 broad categories of medical conditions according to three distinct aspects of social disconnectedness (loneliness, social isolation and low social support), as well as a composite measure, with exploration of sex and age differences and interaction with pre-existing mental disorders.

## Methods

### Study population

We conducted a cohort study of participants from the Danish National Health Survey with linkage to national registers. Every fourth year, the Danish National Health Survey is carried out in five regional stratified random samples and one national random sample (Christensen *et al.*, 2020). Based on the inclusion of questions on social connections, we included 162,604 survey participants: 129,319 from four regions in 2017 (Central Denmark Region, North Denmark Region, Region Zealand and Capital Region of Denmark) and 33,285 from one region in 2013 (Central Denmark Region). Overall, the response rate was 57.5%. We applied inverse probability weights calculated by Statistics Denmark based on national register data to account for non-response and selection probabilities (Christensen *et al.*, 2020). Due to the population-based sample, a minor proportion of the responses (2.1%) were from individuals who participated in both 2013 and 2017. Unique identification numbers from the Danish Civil Registration System (Pedersen, 2011) were used to link the survey data with national registers. After exclusion of 107 individuals (0.07%) with no register linkage at the time of the survey (e.g., due to emigration), the initial study population consisted of the remaining 162,497 individuals. A flowchart delineating the definition of the intial study population is presented in Fig. S1.

### Social connections

Loneliness, social isolation and low social support were assessed using survey data from the Danish National Health Survey. Loneliness refers to an unpleasant emotional experience caused by a perceived lack of social contact (Peplau and Perlman, 1982). Loneliness was assessed with the Danish version of the Three-Item Loneliness Scale (Hughes *et al.*, 2004; Lasgaard, 2007), which provides a score from 3 to 9 with higher scores indicating greater loneliness; a score of 7 or higher was classified as indicating loneliness. The third item was slightly rephrased in 2017 compared to 2013 to enhance correspondence with the definition of loneliness, but the scale has demonstrated good internal consistency at both time points (Laustsen *et al.*, 2023). Social isolation concerns the objective characteristics of a person’s social ties, referring to a limited network or lack of social contact (de Jong-Gierveld *et al.*, 2006). With inspiration from the Berkman-Syme Social Network Index (Berkman and Syme, 1979), social isolation was assessed by quantifying different areas of social contact. Specifically, four indicators of limited social contact were used providing a score ranging from 0 to 4: whether an individual (i) was living alone, (ii) was unemployed and not enrolled in education, (iii) had less than monthly contact with friends and (iv) had less than monthly contact with family outside of the household. A score of 3 or higher was classified as indicating social isolation. Social support encompasses several types of perceived and received support. In this study, we focused on perceived emotional support characterized as the experienced availability of verbal care, acceptance and emotional reciprocity (Cohen Sheldon *et al.* 2000). With inspiration from the MOS Social Support Instrument (Sherbourne and Stewart, 1991), low social support was assessed with the single-item ‘Do you have someone to talk to if you have problems or need for support?’ with four response options: ‘Yes, always’; ‘Yes, mostly’; ‘Yes, sometimes’ and ‘No, never or almost never’. Answers in the two last-mentioned response options were classified as indicating low social support. Lastly, we constructed a composite measure of either loneliness, social isolation and low social support, capturing both the structural and functional aspects of social disconnection.

### Medical conditions

Medical conditions were assessed using 11 broad categories: mental disorders; all-cause dementia; circulatory, endocrine, pulmonary, gastrointestinal, urogenital, musculoskeletal, hematologic and neurologic conditions; and cancer. These 11 broad categories were identified using hospital-based diagnoses from inpatient admissions and outpatient and emergency visits which since 1st of January 1995 have been recorded in the Danish National Patient Registry (Schmidt *et al.*, 2015) and the Danish Psychiatric Central Research Register (Mors *et al.*, 2011), redeemed prescriptions recorded in the Danish National Prescription Registry (Kildemoes *et al.*, 2011), and causes of death recorded in the Danish Register of Causes of Death (Helweg-Larsen, 2011). Except for emergency admissions, general practitioners serve as gatekeepers to inpatient and outpatient hospital care in the Danish healthcare system (Schmidt *et al.*, 2019). Based on prior studies (Elser *et al.*, 2023; Laustsen *et al.*, 2024; Prior *et al.*, 2016), we classified diagnoses (including causes of death) and prescriptions as shown in Table S2, applying the 10th revision of the International Statistical Classification of Diseases and Related Health Problems (ICD-10) and the Anatomical Therapeutic Chemical Classification System (ATC). To detect pre-existing cases (i.e., with onset before the time of survey participation), we obtained information on medical conditions in 18 years preceding survey participation. During follow-up, the onset of a medical condition was defined as the date of the first hospital diagnosis, the date of a repeated redeemed prescription, or the date of death with the medical condition stated as the underlying cause, whichever occurred first. Although data in the Danish Register of Causes of Death is currently only available until 31 December 2020, we allowed for follow-up until the end of data availability in the remaining registers (31 December 2021).

### Covariates

Age, sex (registered legal sex), country of birth (Denmark and Greenland vs. abroad) and linkage to legal parents were obtained from the Danish Civil Registration System (Pedersen, 2011). Highest educational level was obtained from the Population Education Register. Income and wealth were obtained from the Income Statistics Register using the annual disposable equivalized household income and the equivalized household wealth after adjustment for inflation, respectively. For individuals aged 16–29 years, we used their parental highest educational level and an average of parental values for income and wealth. Details are provided in Methods S1.

### Study design

For each category of medical conditions, a cohort design was applied in which individuals without a prior medical condition within the category were followed up from the date of survey participation until the onset of a medical condition within the category, death, emigration or end of data availability (31 December 2021), whichever came first.

### Statistical analysis

To avoid inducing selection bias through exclusion of 20,856 (12.8%) individuals with partially missing register and/or survey data, we conducted multiple imputation by chained equations (Methods S2). To describe baseline characteristics of the cohort, we computed means, standard deviations (SDs) and proportions. All estimates were calculated applying inverse probability of participation weights and pooling multiple imputed data using Rubin’s Rules.

We used Poisson regression models with Taylor-linearized variance estimation and 95% confidence intervals (CIs) to compare the incidence rate of medical conditions in each category between individuals who were social disconnected at baseline with those who were not. Two adjustment models were applied. Model 1 estimated the incidence rate ratio (IRR) after adjustments for demographics (age, sex and year of survey participation). Model 2 additionally adjusted for country of birth and socio-economic resources (educational level, income and wealth) measured in the calendar year preceding survey participation. We additionally estimated the incidence rate difference (IRD) using marginal standardization to compare individuals with versus without the composite measure. Furthermore, we investigated whether these associations varied according to sex and age at baseline (16–65 years, >65 years) in stratified analyses. As sensitivity analyses to assess potential reverse causation, we investigated if similar results were obtained with (i) start of follow-up delayed to 6 months after survey participation and exclusion of individuals who self-reported a medical condition of interest, and (ii) adjustment for self-rated general health at baseline, assuming it acts purely as a confounder. Details are provided in Methods S3.

Subsequently, we repeated the above analysis while exploring deviations from additive interaction between the composite measure of social disconnectedness and pre-existing mental disorders with estimation of the relative excess risk due to interaction (RERI) in all categories of medical conditions, except mental disorders. As mental disorders might impact socio-economic resources, we assessed educational level, income and wealth in the calendar year preceding the diagnosis of a mental disorder. We replicated this adjustment procedure among individuals without a pre-existing mental disorder via the assignment of pseudo-index dates in age- and sex-specific groups with a procedure akin to our prior study (Laustsen *et al.*, 2024). Next, we conducted a subgroup analysis with sex-specific estimates of the interaction. As a sensitivity analysis, we applied a broader definition of mental disorders additionally including self-reported information, redeemed prescriptions of psychopharmaceuticals and consultations with private practicing psychiatrists. Details are provided in Methods S4.

Statistical analyses were conducted in Stata version 18.0 using the svy and mi suite of commands. A preregistered analysis plan and the code used for data management and statistical analysis are available at Open Science Framework (https://osf.io/pycrq).

## Results

Among the 162,497 survey participants, the mean age was 48.3 years (SD 19.1) at survey participation, 87,627 (50.6%) were women, and the number of individuals classified as lonely, socially isolated, and with low social support was 9,808 (7.6%), 4,716 (3.5%) and 21,360 (14.8%), respectively. Co-occurrence of loneliness, social isolation and low social support was similar for men and women as shown in Fig. S2. Baseline characteristics according to social disconnection are shown in Table 1; for instance, the prevalence of pre-existing medical conditions was higher among individuals who were socially disconnected except for urogenital conditions and cancer (additional characteristics of the cohort are shown in Table S3). The number of individuals without a pre-existing medical condition in each category ranged from 110,571 (followed over 575,370 person-years at risk after survey participation) for pulmonary conditions to 161,870 (881,315 person-years at risk) for all-cause dementia. The number of new cases of medical conditions during follow-up ranged from 1,438 for all-cause dementia to 17,818 for musculoskeletal conditions (Table S4). During follow-up, 9,047 individuals died and 2,782 emigrated.

**Table 1.** Baseline characteristics of the cohort in four regions of Denmark, 2013 and 2017

	Lonely: *N* = 9,808 (7.6)	Socially isolated: *N* = 4,716 (3.5)	Low social support: *N* = 21,360 (14.8)	Neither lonely, socially isolated or low social support: *N* = 134,232 (80.0)
Age, mean (SD)	43.3 (25.9)	64.4 (27.2)	48.1 (25.0)	48.2 (23.1)
Women, *N* (%)	5,831 (55.2)	2,186 (44.5)	10,434 (45.0)	73,180 (51.3)
Survey participation in 2013 as opposed to 2017, *N* (%)	1,245 (13.6)	901 (19.3)	4,232 (20.1)	27,966 (22.2)
Born abroad, *N* (%)	1,553 (22.9)	472 (14.6)	2,974 (21.3)	8,432 (10.2)
Educational level*				
Lowest (ISCED 0–2), *N* (%)	3,043 (33.7)	2,123 (50.0)	6,062 (31.1)	28,947 (23.8)
Middle (ISCED 3–5), *N* (%)	4,648 (45.5)	1,909 (37.4)	10,594 (47.5)	65,813 (48.0)
Highest (ISCED 6–8), *N* (%)	2,117 (20.8)	685 (12.6)	4,704 (21.4)	39,471 (28.2)
Annual disposable equivalized household income* (1,000 DKK), mean (SD)	238.8 (213.2)	192.6 (115.7)	249.9 (205.9)	296.1 (315.1)
Equivalized household wealth* (1,000 DKK), mean (SD)	315.6 (2,072.7)	516.6 (2,296.5)	451.8 (2,037.5)	573.4 (3,757.3)
The Three-Item Loneliness Scale, mean (SD)	7.8 (1.2)	5.4 (2.8)	5.4 (2.6)	3.6 (1.2)
The adapted Social Isolation Index				
Living alone, *N* (%)	3,707 (40.5)	3,552 (79.2)	6,239 (33.5)	21,314 (19.1)
Out of employment and not enrolled in education, *N* (%)	4,572 (44.8)	4,479 (93.7)	8,862 (40.0)	46,494 (30.9)
Less than monthly contact with friends, *N* (%)	2,505 (24.9)	3,472 (73.2)	4,303 (20.4)	7,210 (5.1)
Less than monthly contact with family, *N* (%)	1,894 (20.1)	3,165 (66.3)	3,678 (18.8)	6,122 (5.0)
The social support item				
Social support never or almost never available, *N* (%)	1,945 (20.5)	959 (22.1)	6,638 (32.3)	0 (0)
Social support sometimes available, *N* (%)	3,247 (32.5)	1,063 (23.2)	14,722 (67.7)	0 (0)
Social support mostly available, *N* (%)	2,796 (28.4)	1,241 (25.6)	0 (0)	39,888 (30.2)
Social support always available, *N* (%)	1,820 (18.6)	1,453 (29.1)	0 (0)	94,344 (69.8)
Pre-existing mental disorder, *N* (%)	2,313 (25.4)	807 (20.1)	2,544 (13.8)	6,478 (5.7)
Pre-existing all-cause dementia, *N* (%)	127 (1.1)	109 (2.2)	120 (0.5)	380 (0.3)
Pre-existing circulatory condition, *N* (%)	3,364 (28.8)	3,075 (60.6)	8,080 (32.1)	47,849 (29.7)
Pre-existing endocrine condition, *N* (%)	1,407 (12.4)	1,102 (22.5)	2,987 (12.2)	15,647 (10.2)
Pre-existing pulmonary condition or allergy, *N* (%)	3,637 (34.6)	1,710 (35.1)	7,136 (32.0)	42,332 (30.5)
Pre-existing gastrointestinal condition, *N* (%)	699 (6.5)	563 (11.6)	1,412 (6.1)	7,169 (4.7)
Pre-existing urogenital condition, *N* (%)	418 (3.4)	546 (10.5)	1,057 (4.2)	5,916 (3.6)
Pre-existing musculoskeletal condition, *N* (%)	3,698 (33.2)	2,539 (52.5)	7,654 (31.9)	40,854 (26.7)
Pre-existing hematologic condition, *N* (%)	301 (2.9)	262 (5.5)	544 (2.5)	2,365 (1.7)
Pre-existing neurologic condition, *N* (%)	2,347 (20.6)	1,758 (35.1)	4,796 (19.7)	26,391 (17.2)
Pre-existing cancer, *N* (%)	494 (3.9)	536 (10.0)	1,215 (4.7)	8,546 (5.3)

* C.f. data from the preceding calendar year.

Missing data were imputed using multiple imputation by chained equations. Absolute numbers are unweighted, whereas means, standard deviations and percentages are weighted based on register data to represent the population of the included regions in 2013 and 2017. Note that loneliness, social isolation and low social support are not mutually exclusive; therefore, the percentages in the top row does not sum up to 1.

**Figure 1.**
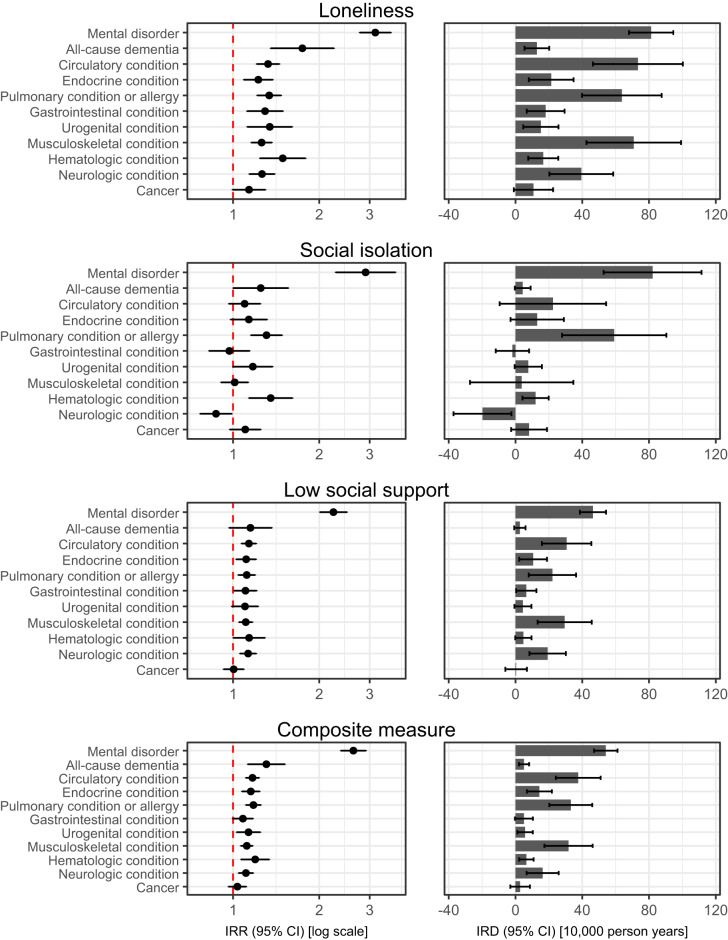
Social disconnectedness and rates of subsequent medical conditions in 11 broad categories in four regions of Denmark, 2013–2021.

Figure 1 provides the relative and absolute differences in incidence rates of medical conditions according to social disconnectedness based on Model 2 (adjusted for demographics, country of birth and socio-economic resources). Overall, the median and interquartile range (IQR) of the IRRs of medical conditions for loneliness, social isolation, low social support and the composite measure were respectively 1.32 (IQR, 1.26–1.49), 1.14 (IQR, 1.01–1.31), 1.12 (IQR, 1.10–1.14) and 1.15 (IQR, 1.11–1.19). Individuals who were lonely had higher incidence rates in all 11 categories of which the estimate for cancer was also consistent with a lower rate (IRR, 1.14 [95% CI, 1.00–1.30]). Individuals who were socially isolated had higher incidence rates in nine categories of which the estimates in six categories were also consistent with a lower rate and had lower incidence rates of gastrointestinal and neurologic conditions of which the estimate for the former was also consistent with a higher rate (IRR, respectively 0.97 [95% CI, 0.82–1.14] and 0.87 [95% CI, 0.77–0.99]). Individuals with low social support had higher incidence rates in all 11 categories of which the estimates in four categories were also consistent with a lower rate. The overall strongest associations were found for mental disorders, which for loneliness, social isolation, low social support and the composite measure provided an IRR of respectively 3.14 (95% CI, 2.77–3.56), 2.90 (95% CI, 2.28–3.69), 2.24 (95% CI, 2.01–2.50) and 2.63 (95% CI, 2.38–2.91), corresponding to an IRD of respectively 81 (95% CI, 68–95), 82 (95% CI, 53–111), 46 (95% CI, 39–54) and 54 (95% CI, 47–61) cases per 10,000 person-years. The overall weakest associations were found for cancer. Estimates from Model 1 were substantially similar and are provided in Table S5.

CI: confidence interval; IRD: incidence rate difference; IRR: incidence rate ratio. IRRs are displayed on a logarithmic scale, whereas IRDs are displayed on a linear scale. Missing data were imputed using multiple imputation by chained equations, and the results are weighted based on register data to represent the population of the included regions in 2013 and 2017. IRDs are calculated using marginal standardization to individuals with versus without at least one of loneliness, social isolation and low social support. All estimates are adjusted for age, sex, year of survey participation, country of birth, educational level, income and wealth (Model 2) and are provided in Table S5.

**Figure 2.**
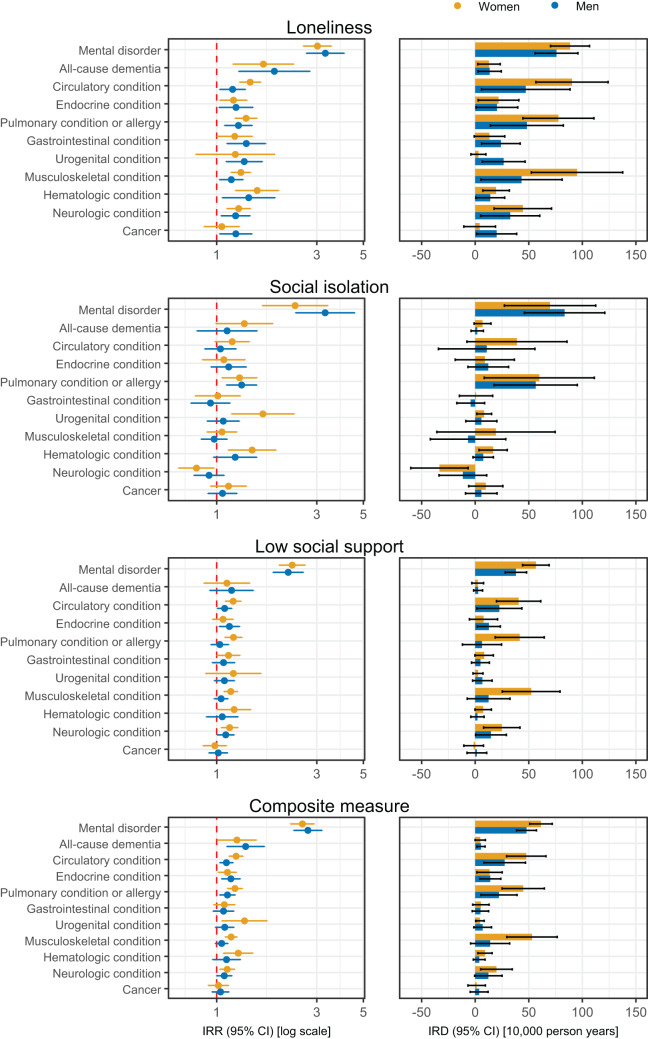
Social disconnectedness and sex-stratified rates of subsequent medical conditions in 11 broad categories in four regions of Denmark, 2013–2021.

**Figure 3.**
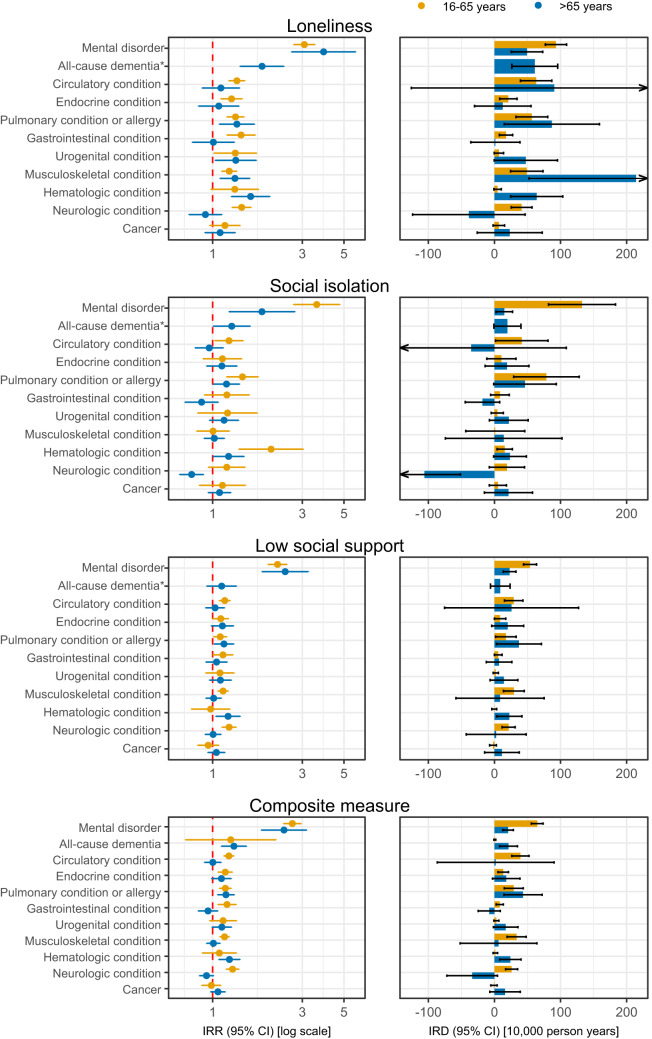
Social disconnectedness and age-stratified rates of subsequent medical conditions in 11 broad categories in four regions of Denmark, 2013–2021.

Figures 2 and 3 provides sex- and age-stratified differences in incidence rates of medical conditions according to social disconnectedness. In the sex-stratified analysis, the median IRR of medical conditions for the composite measure was 1.22 (IQR, 1.12–1.27) for women and 1.11 (IQR, 1.08–1.17) for men, and considerable sex differences were observed for some medical conditions in the relative or absolute estimates. For instance, the IRR for urogenital conditions according to social isolation was 1.66 (95% CI, 1.09–2.23) for women and 1.08 (95% CI, 0.89–1.27) for men, whereas the IRD for musculoskeletal conditions according to loneliness was 98 (95% CI, 56–141) cases per 10,000 person-years for women and 47 (95% CI, 7–87) for men. In the age-stratified analysis, the median IRR of medical conditions for the composite measure was 1.17 (IQR, 1.14–1.27) for individuals aged 16–65 years and 1.11 (IQR, 1.00–1.23) for individuals aged above 65 years. For social isolation, the results indicated greater relative differences for younger individuals. For instance, the IRR for mental disorders according to social isolation was 3.58 (95% CI, 2.69–4.76) for individuals aged 16–65 years and 1.83 (95% CI, 1.22–2.74) for individuals aged above 65 years. Furthermore, major differences in some absolute estimates were observed. For instance, the IRD for musculoskeletal conditions according to loneliness was 214 (95% CI, 52–376) cases per 10,000 person-years for individuals aged 16–65 years and 49 (95%, 24–73) for individuals aged above 65 years. The sensitivity analysis with delayed start of follow-up by 6 months and exclusion of individuals with a self-reported medical condition provided similar IRRs, but attenuated IRDs, especially for mental disorders. Likewise, the sensitivity analysis with adjustment for self-rated general health provided attenuated IRRs (Fig. S3).

CI: confidence interval; IRD: incidence rate difference; IRR: incidence rate ratio. IRRs are displayed on a logarithmic scale, whereas IRDs are displayed on a linear scale. Missing data were imputed using multiple imputation by chained equations, and the results are weighted based on register data to represent the population of the included regions in 2013 and 2017. IRDs are calculated using marginal standardization to individuals with versus without at least one of loneliness, social isolation and low social support. All estimates are adjusted for age, year of survey participation, country of birth, educational level, income and wealth (Model 2) and are provided in Table S6.

CI: confidence interval; IRD: incidence rate difference; IRR: incidence rate ratio. IRRs are displayed on a logarithmic scale, whereas IRDs are displayed on a linear scale. Missing data were imputed using multiple imputation by chained equations, and the results are weighted based on register data to represent the population of the included regions in 2013 and 2017. IRDs are calculated using marginal standardization to individuals with versus without at least one of loneliness, social isolation and low social support. All estimates are adjusted for sex, age, year of survey participation, country of birth, educational level, income and wealth (Model 2) and are provided in Table S7.

*Results for each separate aspect of social disconnectedness are not provided among individuals aged 16–65 years due to few cases of all-cause dementia in this age group.

**Figure 4.**
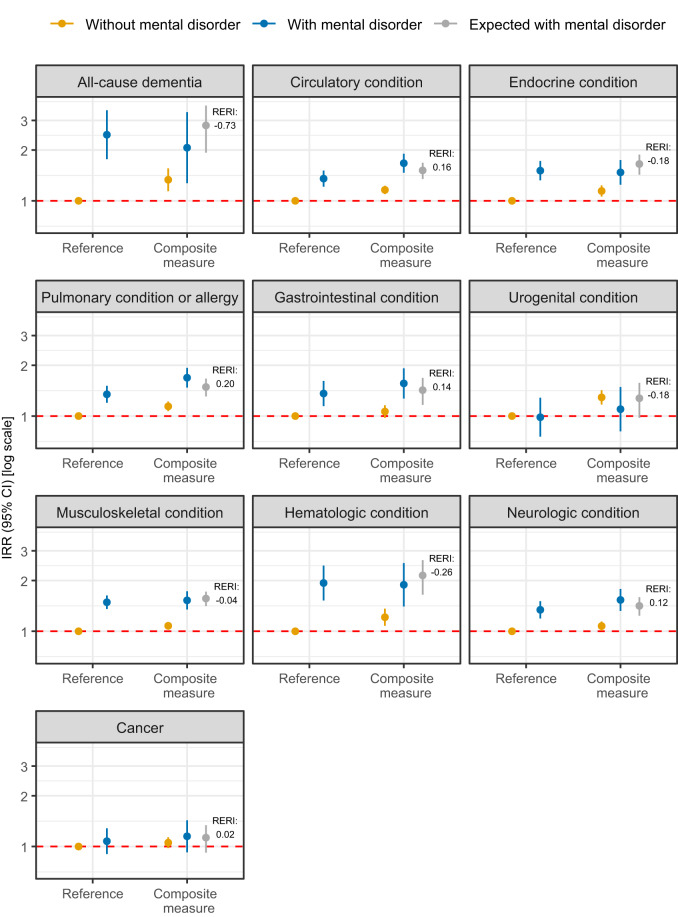
Interaction between social disconnectedness and pre-existing mental disorders on rates of subsequent medical conditions in 10 broad categories in four regions of Denmark, 2013–2021.

Findings from the analysis of interaction with pre-existing mental disorders are provided in Figure. 4. The median RERI was −0.01 (IQR, −0.18–0.14). In five out of ten categories of medical conditions, the incidence rate of a medical condition among those with both a pre-existing mental disorder and the composite measure of social disconnectedness was below that expected based on additive interaction. However, these findings were subject to substantial uncertainty, and the direction of any deviation from additive interaction was unclear in all 10 categories. Furthermore, no consistent differences in the deviations from additive interaction could be identified in the sex-stratified analysis (Fig. S4). The sensitivity analysis concerning the operationalization of pre-existing mental disorders provided attenuated IRRs for pre-existing mental disorders and subsequent medical conditions, but similar results regarding deviations from additive interaction (Fig. S5).

CI: confidence interval; IRR: incident rate ratio; RERI: relative excess Risk due to interaction. IRRs are displayed on a logarithmic scale. Missing data were imputed using multiple imputation by chained equations, and the results are weighted based on register data to represent the population of the included regions in 2013 and 2017. The estimates of expected with a mental disorder are calculated based on additive interaction between mental disorders and the composite measure (at least one of loneliness, social isolation and low social support). All estimates are adjusted for age, sex, year of survey participation, country of birth, educational level, income and wealth (Model 2) and are provided in Table S8.

## Discussion

In this population-based cohort study based on 162,497 participants from the Danish National Health Survey, we quantified differences in the incidence rates of medical conditions according to social disconnectedness. In general, individuals who were socially disconnected, especially lonely, had a higher incidence rate of the investigated categories of medical conditions. However, we also observed a lower incidence rate of gastrointestinal and neurologic conditions for individuals who were socially isolated. The overall greatest relative and absolute differences in incidence rates were observed for mental disorders, whereas the lowest were observed for cancer. We found sex and age differences in some absolute and relative estimates, but no substantial deviations from additive interaction with pre-existing mental disorders. These results were robust to the performed sensitivity analyses although attenuated IRRs were found after adjustment for self-rated general health.

### Comparison to prior studies

We generally obtained similar results compared to the relative risks in previous systematic reviews (Table S1) although some of our estimates are slightly greater for individuals who were lonely and slightly lower for individuals who were socially isolated. The former could be due to our definition of loneliness which corresponds to the most conservative applied dichotomization of the Three-Item Loneliness Scale (Nielsen *et al.*, 2021), and the latter could be owing to differences in the applied measures as there is no agreement on a standardized method for measuring social isolation (Prohaska *et al.*, 2020). Nevertheless, our results indicate that loneliness is a stronger determinant of medical conditions than social isolation and low social support, contrary to prior evidence (Leigh-Hunt *et al.*, 2017). Interestingly, our findings of no substantial deviations from additive interaction with pre-existing mental disorders differ from a Norwegian population-based cohort study based on 25,964 individuals which indicated greater age- and sex-adjusted incidence rates of venous thromboembolism than expected among individuals with concurrent loneliness and depression (Enga *et al.*, 2012). Possibly, the accessibility of prior studies reporting no substantial interaction could have been affected by publication bias.

### Strengths and limitations

Our study benefitted from a large population-based sample with linkage of survey and register data, imputation of partially missing data and application of inverse probability weights to account for potential selection bias. With data from the Danish National Health Survey, we were able to apply a validated measure of loneliness (Lasgaard, 2007) and a measure of social isolation with inspiration from the Berkman-Syme Social Network Index (Berkman and Syme, 1979). Furthermore, we used register data on medical conditions to circumvent loss to follow-up and applied a washout period of 18 years to exclude pre-existing conditions. Not least, the inclusion of indicators to capture three distinct aspects of social disconnectedness and the assessment of medical conditions in 11 different categories enabled a more comprehensive approach with a better basis for comparisons across different indicators and different categories of medical conditions.

Our study also has important limitation. Certainty regarding the representativeness of the study participants is not possible although the inverse probability weights account well for primary healthcare utilization (Jensen *et al.*, 2022) and mental disorders (Momen *et al.*, 2022). Survey participation could be lower among socially disconnected individuals with a predisposition to development of a medical condition, leading to an underestimation of relative and absolute differences. Additionally, our social isolation index did not account for associational activities and voluntary work, and the measure of social support only examined perceived emotional support (Cohen *et al.*, 2000). Furthermore, the reliance on register-based diagnoses and redeemed prescriptions has probably led to an underestimation of the absolute differences as some medical conditions can be diagnosed and treated by general practitioners. Likewise, the primary operationalization of pre-existing mental disorders based on psychiatric hospital diagnoses will only capture individuals with a high severity of common mental disorders such as depression (Weye *et al.*, 2023) as individuals treated by general practitioners and private practice psychiatrists and untreated individuals are not included. Outcome misclassification due to diagnostic delay and undiagnosed illness could also lead to underestimation of medical conditions, or if such misclassification depends on social disconnectedness, it could lead to bias in an unpredictable direction. Not least, it is essential to account for variations in healthcare systems and cultural contexts when applying these findings, particularly the absolute estimates, to other settings (Luhmann *et al.*, 2023).

### Potential explanations and implications

The finding of a lower incidence rate of neurologic conditions among individuals who were socially isolated is unexpected, but several explanations could be applied. Two of these could be outcome misclassification and reverse causation, e.g., due to a diagnostic delay for vision and hearing problems among elderly individuals. The remaining findings of higher incidence rates of medical conditions among individuals who were socially disconnected point to several possible explanations as a topic for future investigations. Our findings could also partially be explained by undetected medical conditions causing social disconnection as the absence of repeated measurements of social disconnectedness complicates the elimination of reverse causation. This could especially be the case for loneliness and subsequent mental disorders such as depression as they share a high degree of symptomatology (Cacioppo *et al.,* 2006). However, we are not able to distinguish between confounding and mediating effects of baseline measurements such as self-rated health as we cannot ascertain whether they preceded or followed social disconnection. Furthermore, based on the conceptual model suggested by Berkman *et al.* (2000), social connections could influence health through health behaviour pathways, psychological pathways and physiological pathways. Health behaviour pathways – e.g., smoking, diet and physical activity – could impact the development of cardiovascular and pulmonary diseases. Psychological pathways – e.g., coping, self-efficacy and distress – could impact the development of mental disorders and musculoskeletal pain. Physiological pathways – e.g., HPA axis response, allostatic load and immune system function – could impact biological aging and, in turn, the development of dementia, diabetes and migraine. These pathways might differ for the three aspects of social disconnectedness and might be intertwined. For instance, coping could also impact the ability to prevent occurring symptoms from developing into a medical condition in need of hospital-based treatment. Our findings support the notion that social connections are vital for maintaining health, thus emphasizing the importance of addressing loneliness for both physical and mental health.

Our sex- and age-stratified results with differences in the relative estimates suggest that the strength of the hypothesized pathways could vary according to sex or age for some medical conditions, and that social isolation may be a more potent indicator in the non-elderly population. Differences in the absolute estimates according to sex and age might also be attributable to underlying sex- and age-differences in the incidence rates. Taken together, these age- and sex-stratified results may be of significance for mapping group-specific preventative needs and guiding health practitioners aiming to reduce the disease burden in specific subgroup. Furthermore, the result of no substantial deviations from additive interaction with pre-existing mental disorders are unexpected given our prior findings on mortality (Laustsen *et al.*, 2024). Although these findings on mortality cannot be explained by a higher incidence of medical conditions beyond that expected based on additive interaction, we were not able to explore the severity or treatment of medical conditions nor results for different diagnostic groups of pre-existing mental disorders.

## Conclusions

Our results expand existing evidence linking social disconnectedness to elevated risks of mental disorders, dementia, circulatory conditions and musculoskeletal conditions (Mann *et al.*, 2022; Valtorta *et al.*, 2016; Wang *et al.*, 2023b; Yang *et al.*, 2023). Notably, we additionally found higher incidence rates of endocrine, pulmonary, gastrointestinal, urogenital, hematologic, and neurologic conditions and cancer although the estimates for cancer were also consistent with lower rates. Contrary to previous evidence, our findings suggest that loneliness is a stronger determinant for subsequent medical conditions than social isolation and low social support. We found sex and age differences in some relative and absolute estimates, but no substantial deviations from additive interaction with pre-existing mental disorders.

## Supporting information

Laustsen et al. supplementary material Laustsen et al. supplementary material

## Data Availability

Data presented in this study were obtained from Danish registries and regions participating in the Danish National Health Survey. Owing to data protection rules, we are not allowed to share individual-level data. Other researchers who fulfil the requirements set by the data providers may gain access to the data through Statistics Denmark, the Danish Health Data Authority and/or the Danish regions (Central Denmark Region, North Denmark Region, Region Zealand and Capital Region of Denmark). A preregistered analysis plan and all statistical code from the main analysis are available at Open Science Framework (https://osf.io/pycrq).

## Supplementary material

The supplementary material for this article can be found at https://doi.org/10.1017/S2045796024000829.

## References

[ref1] Berkman LF, Glass T, Brissette I and Seeman TE (2000) From social integration to health: Durkheim in the new millennium. *Social Science & Medicine (1982)* 51(6), 843–857. doi:10.1016/s0277-9536(00)00065-4. 10972429

[ref2] Berkman LF and Syme SL (1979) Social networks, host resistance, and mortality: A nine-year follow-up study of Alameda County residents. *American Journal of Epidemiology* 109(2), 186–204. doi:10.1093/oxfordjournals.aje.a112674. 425958

[ref3] Cacioppo JT, Hughes ME, Waite LJ, Hawkley LC and Thisted RA (2006) Loneliness as a specific risk factor for depressive symptoms: Cross-sectional and longitudinal analyses. *Psychology and Aging* 21(1), 140–151. doi:10.1037/0882-7974.21.1.140. 16594799

[ref4] Christensen AI, Lau CJ, Kristensen PL, Johnsen SB, Wingstrand A, Friis K, Davidsen M and Andreasen AH (2020) The Danish National Health Survey: Study design, response rate and respondent characteristics in 2010, 2013 and 2017. *Scandinavian Journal of Public Health* 50(2), 180–188. doi:10.1177/1403494820966534. 33161874

[ref5] Cohen S, Brittney L and Gottlieb Benjamin H (2000) *Social Support Measurement and Intervention: A Guide for Health and Social Scientists*. Oxford: Oxford University Press.

[ref6] de Jong-Gierveld J, van Tilburg T and Dykstra PA (2006) Loneliness and social isolation. In Vangelisti A and Perlman D (eds), *The Cambridge Handbook of Personal Relationships*. Cambridge: Cambridge University Press, 485–500.

[ref7] Elser H, Horváth-Puhó E, Gradus JL, Smith ML, Lash TL, Glymour MM, Sørensen HT and Henderson VW (2023) Association of early-, middle-, and late-life depression with incident dementia in a Danish cohort. *JAMA Neurology* 80(9), 949–958. doi:10.1001/jamaneurol.2023.2309. 37486689 PMC10366950

[ref8] Enga KF, Brækkan SK, Hansen-Krone IJ and Hansen J-B (2012) Emotional states and future risk of venous thromboembolism: The Tromsø Study. *Thrombosis and Haemostasis* 107(3), 485–493. doi:10.1160/TH11-09-0667. 22318455

[ref9] Fang B, Yang S, Liu H, Zhang Y, Xu R and Chen G (2019) Association between depression and subsequent peptic ulcer occurrence among older people living alone: A prospective study investigating the role of change in social engagement. *Journal of Psychosomatic Research* 122, 94–103. doi:10.1016/j.jpsychores.2019.04.002. 30975521

[ref10] Guo L, Wang W, Shi J, Zheng X, Hua Y and Lu C (2023) Evaluation of social isolation trajectories and incident cardiovascular disease among middle-aged and older adults in China: National cohort study. *JMIR Public Health and Surveillance* 9, e45677. doi:10.2196/45677. PMC10365588 37389914

[ref11] Helweg-Larsen K (2011) The Danish register of causes of death. *Scandinavian Journal of Public Health* 39(7 Suppl), 26–29. doi:10.1177/1403494811399958. 21775346

[ref12] Hughes ME, Waite LJ, Hawkley LC and Cacioppo JT (2004) A short scale for measuring loneliness in large surveys: Results from two population-based studies. *Research on Aging* 26(6), 655–672. doi:10.1177/0164027504268574. 18504506 PMC2394670

[ref13] Jensen HAR, Lau CJ, Davidsen M, Feveile HB, Christensen AI and Ekholm O (2022) The impact of non-response weighting in health surveys for estimates on primary health care utilization. *European Journal of Public Health* 32(3), 450–455. doi:10.1093/eurpub/ckac032. 35373254 PMC9159316

[ref14] Kildemoes HW, Sørensen HT and Hallas J (2011) The Danish national prescription registry. *Scandinavian Journal of Public Health* 39(7 Suppl), 38–41. doi:10.1177/1403494810394717. 21775349

[ref15] Lasgaard M (2007) Reliability and validity of the Danish version of the UCLA loneliness scale. *Personality and Individual Differences* 42, 1359–1366. doi:10.1016/j.paid.2006.10.013.

[ref16] Laustsen LM, Christiansen J, Maindal HT, Plana-Ripoll O and Lasgaard M (2023) The longitudinal relation between loneliness and perceived stress: A structural equation modelling analysis of 10,159 individuals. *Scandinavian Journal of Public Health* 14034948231151716. doi:10.1177/14034948231151716. 36794680

[ref17] Laustsen LM, Ejlskov L, Chen D, Lasgaard M, Gradus JL, Østergaard SD, Grønkjær MS and Plana-Ripoll O (2024) Interaction between mental disorders and social disconnectedness on mortality: A population-based cohort study. *The British Journal of Psychiatry* 225(1), 282–289. doi:10.1192/bjp.2024.68. 38708564

[ref18] Leigh-Hunt N, Bagguley D, Bash K, Turner V, Turnbull S, Valtorta N and Caan W (2017) An overview of systematic reviews on the public health consequences of social isolation and loneliness. *Public Health* 152, 157–171. doi:10.1016/j.puhe.2017.07.035. 28915435

[ref19] Liang Y Yan, Yannis Yan Liang, Mingqing Zhou, Yu He, Weijie Zhang, Qiqi Wu, Tong Luo, Jun Zhang, Fujun Jia, Lu Qi, Sizhi Ai and Jihui Zhang (2024). Observational and genetic evidence disagree on the association between loneliness and risk of multiple diseases. Nat Hum Behav, 8(11), 2209–2221. doi:10.1038/s41562-024-01970-0. 39284978 PMC11576506

[ref20] Luhmann M, Buecker S and Rüsberg M (2023) Loneliness across time and space. *Nature Reviews Psychology* 2(1), 9–23. doi:10.1038/s44159-022-00124-1. PMC9640887 36406179

[ref21] Mann F, Wang J, Pearce E, Ma R, Schlief M, Lloyd-Evans B, Ikhtabi S and Johnson S (2022) Loneliness and the onset of new mental health problems in the general population. *Social Psychiatry & Psychiatric Epidemiology* 57(11), 2161–2178. doi:10.1007/s00127-022-02261-7. 35583561 PMC9636084

[ref22] Momen NC, Lasgaard M, Weye N, Edwards J, McGrath J and Plana-Ripoll O (2022) Representativeness of survey participants in relation to mental disorders: A linkage between national registers and a population-representative survey. *International Journal of Population Data Science* 7(4). doi:10.23889/ijpds.v7i4.1759. PMC10161967 37152406

[ref23] Momen NC, Plana-Ripoll O, Agerbo E, Benros ME, Børglum AD, Christensen MK, Dalsgaard S, Degenhardt L, de Jonge P, Debost J-CPG, Fenger-Grøn M, Gunn JM, Iburg KM, Kessing LV, Kessler RC, Laursen TM, Lim CCW, Mors O, Mortensen PB, Musliner KL, Nordentoft M, Pedersen CB, Petersen LV, Ribe AR, Roest AM, Saha S, Schork AJ, Scott KM, Sievert C, Sørensen HJ, Stedman TJ, Vestergaard M, Vilhjalmsson B, Werge T, Weye N, Whiteford HA, Prior A and McGrath JJ (2020) Association between mental disorders and subsequent medical conditions. *New England Journal of Medicine* 382(18), 1721–1731. doi:10.1056/NEJMoa1915784. 32348643 PMC7261506

[ref24] Mors O, Perto GP and Mortensen PB (2011) The Danish psychiatric central research register. *Scandinavian Journal of Public Health* 39(7 Suppl), 54–57. doi:10.1177/1403494810395825. 21775352

[ref25] Nielsen T, Friderichsen I and Rayce S (2021) Classification of loneliness using the T-ILS: Is it as simple as it seems? *Scandinavian Journal of Psychology* 62, 104–115. doi:10.1111/sjop.12697. 33320357

[ref26] Pearce E, Birken M, Pais S, Tamworth M, Ng Y, Wang J, Chipp B, Crane E, Schlief M, Yang J, Stamos A, Cheng LK, Condon M, Lloyd-Evans B, Kirkbride JB, Osborn D, Pitman A and Johnson S (2023) Associations between constructs related to social relationships and mental health conditions and symptoms: An umbrella review. *BMC Psychiatry.* 23(1). doi:10.1186/s12888-023-05069-0. PMC10478264 37667255

[ref27] Pedersen CB (2011) The Danish civil registration system. *Scandinavian Journal of Public Health* 39(7 Suppl), 22–25. doi:10.1177/1403494810387965. 21775345

[ref28] Peplau AL and Perlman D (1982) Perspectives on loneliness. In Peplau, AL and Perlman, D (eds), *Loneliness: A Sourcebook of Current Theory, Research, and Therapy*. New York, NY: Wiley, 1–18.

[ref29] Prior A, Fenger-Grøn M, Larsen KK, Larsen FB, Robinson KM, Nielsen MG, Christensen KS, Mercer SW and Vestergaard M (2016) The association between perceived stress and mortality among people with multimorbidity: A prospective population-based cohort study. *American Journal of Epidemiology* 184(3), 199–210. doi:10.1093/aje/kwv324. 27407085

[ref30] Prohaska T, Burholt V, Burns A, Golden J, Hawkley L, Lawlor B, Leavey G, Lubben J, O’Sullivan R, Perissinotto C, van Tilburg V, Tully M, Victor C and Fried L (2020) Consensus statement: Loneliness in older adults, the 21st century social determinant of health? *BMJ Open* 10(8), e034967. doi:10.1136/bmjopen-2019-034967. PMC7422633 32788184

[ref31] Schmidt M, Schmidt SAJ, Adelborg K, Sundbøll J, Laugesen K, Ehrenstein V and Sørensen HT (2019) The Danish health care system and epidemiological research: From health care contacts to database records. *Clinical Epidemiology* 11, 563–591. doi:10.2147/CLEP.S179083. 31372058 PMC6634267

[ref32] Schmidt M, Schmidt SAJ, Sandegaard JL, Ehrenstein V, Pedersen L and Sørensen HT (2015) The Danish national patient registry: A review of content, data quality, and research potential. *Clinical Epidemiology* 7, 449–490. doi:10.2147/CLEP.S91125. 26604824 PMC4655913

[ref33] Sherbourne CD and Stewart AL (1991) The MOS social support survey. *Social Science & Medicine (1982)* 32(6), 705–714. doi:10.1016/0277-9536(91)90150-b. 2035047

[ref34] Valtorta NK, Kanaan M, Gilbody S, Ronzi S and Hanratty B (2016) Loneliness and social isolation as risk factors for coronary heart disease and stroke: Systematic review and meta-analysis of longitudinal observational studies. *Heart* 102(13), 1009–1016. doi:10.1136/heartjnl-2015-308790. 27091846 PMC4941172

[ref35] Wang F, Gao Y, Han Z, Yu Y, Long Z, Jiang X, Wu Y, Pei B, Cao Y, Ye J, Wang M and Zhao Y (2023a) A systematic review and meta-analysis of 90 cohort studies of social isolation, loneliness and mortality. *Nature Human Behaviour* 1–13. doi:10.1038/s41562-023-01617-6. 37337095

[ref36] Wang S, Molassiotis A, Guo C, Leung ISH and Leung AYM (2023b) Association between social integration and risk of dementia: A systematic review and meta-analysis of longitudinal studies. *Journal of the American Geriatrics Society* 71(2), 632–645. doi:10.1111/jgs.18094. 36307921

[ref37] Weye N, McGrath JJ, Lasgaard M, Momen NC, Knudsen AK, Musliner K and Plana-Ripoll O (2023) Agreement between survey- and register-based measures of depression in Denmark. *Acta Psychiatrica Scandinavica* 147(6), 581–592. doi:10.1111/acps.13555. 37057386

[ref38] Yang J, Huang J, Yang X, Li S, Wu X and Ma X (2023) The association of living alone and social isolation with sarcopenia: A systematic review and meta-analysis. *Ageing Research Reviews* 91, 102043. doi:10.1016/j.arr.2023.102043. 37647996

