# Supplementary material for: Social disconnectedness, subsequent medical conditions, and, the role of pre-existing mental disorders: a population-based cohort study
Source: Epidemiol Psychiatr Sci. 2024 Dec 23;33:e81. doi: 10.1017/S2045796024000829 (PMC11735122; doi:10.1017/S2045796024000829)
Supplement: Laustsen et al. supplementary material [file S2045796024000829sup001.pdf]

Supplementary appendix

This appendix has been provided by the authors to give readers additional information about the following paper:

Social disconnectedness, subsequent medical conditions, and the role of pre-existing mental disorders: a population-based cohort study

Authors: Lisbeth Mølgaard Laustsen, Mathias Lasgaard, Natalie C. Momen, Danni Chen, Jaimie L. Gradus, Marie Stjerne Grønkjær, Martin Mejlby Jensen, Oleguer Plana-Ripoll

Contents

Supplementary Methods **2**

Methods S1. Definition of covariates 2

Methods S2. Multiple imputation 2

Methods S3. Main analysis 7

Methods S4. Interaction analysis 8

Supplementary Figures **9**

Fig. S1. Flowchart depicting the initial study population in four regions of Denmark, 2013 and 2017 9

Fig. S2. Venn diagrams of the distribution of loneliness, social isolation, and low social support among women and men in four regions of Denmark, 2013 and 2017 9

Fig. S3. Sensitivity analysis of social disconnectedness and rates of subsequent medical conditions in 11 broad categories in four regions of Denmark, 2013-2021 10

Fig. S4. Interaction between social disconnectedness and pre-existing mental disorders on sex-specific rates of subsequent medical conditions in 10 broad categories in four regions of Denmark, 2013-2021 11

Fig. S5. Sensitivity analysis on interaction between social disconnectedness and pre-existing mental disorders on rates of subsequent medical conditions in 10 broad categories in four regions of Denmark, 2013-2021 ... 12

Supplementary Tables **13**

Table S1: Overview of systematic reviews and meta-analyses of longitudinal studies assessing social disconnectedness and subsequent medical conditions 13

Table S2. List of diagnosis and prescription codes in each medical condition category 15

Table S3. Additional characteristics of the cohort in four regions of Denmark, 2013 and 2017 17

Table S4. Persons at risk at start of follow-up and cases during follow-up in the total cohort of 162,497 individuals in four regions of Denmark, 2013-2021 18

Table S5. Social disconnectedness and rates of subsequent medical conditions in 11 broad categories in four regions of Denmark, 2013-2021 19

Table S6. Social disconnectedness and sex-stratified rates of subsequent medical conditions in 11 broad categories in four regions of Denmark, 2013-2021 20

Table S7. Social disconnectedness and age-stratified rates of subsequent medical conditions in 11 broad categories in four regions of Denmark, 2013-2021 21

Table S8. Interaction between social disconnectedness and pre-existing mental disorders on rates of subsequent medical conditions in 10 broad categories in four regions of Denmark, 2013-2021 22

References **23**

Supplementary Methods

Methods S1. Definition of covariates

Educational level

We applied the International Standard Classification of Education (ISCED) (1) and classified the highest educational level as a bachelor's degree or higher (ISCED levels 6–8); the middle educational level as upper secondary school, vocational education, or short-cycle tertiary education (ISCED levels 3–5); and the lowest educational level as up to secondary school (ISCED levels 0–2).

Income and wealth

Income and wealth were both based on household values and equivalised using OECD's modified equivalence scale (2). Values were standardized to those in 2016 using the new gross domestic product deflator from the World Bank (worldbank.org) to account for inflation. Disposable income included any registered work income, capital income, and transfers of public benefits after deduction of tax and, to allow for comparison between homeowners and tenants, the estimated rental value of housing for homeowners after exclusion of their interest expenses (3). Wealth included all assets and debts in properties, financial institutions, investment deposits, and credit unions, but did not include pension assets, debt owed to private individuals, investments outside deposits, cash holdings, and other assets such as cars or yachts (4).

Methods S2. Multiple imputation

The distribution of missing values in survey and register data is shown in Methods Table 1. To determine whether a complete case analysis would entail risk of bias (5,6), it is important to explore whether individuals with partly missing survey data were more likely to experience the outcome. As an example, we have displayed the results for circulatory conditions (Methods Table 2). Since this was the case, we conducted multiple imputation by chained equations (MICE) as this will reduce this bias if the included auxiliary variables can predict the missing values. Descriptive characteristics of individuals with missing data are provided in Methods Table 3; noteworthy, individuals with missing register data were more likely to be born abroad, whereas individuals with missing survey data were slightly older and more likely to be out of employment and live alone.

We imputed survey and register data in separate imputation models, each conducting 15 imputations with I burn-in of 10. All missing values were imputed with predictive mean matching (PMM) using a recommended donor pool of 10 observations (7). Survey data on specific scores/responses on loneliness, social isolation, and social support were imputed using auxiliary variables as shown in Methods Table 4. Register data on educational level, income, wealth, and country of birth were imputed for values 1) preceding survey participation and 2) preceding the index or pseudo-index date, using auxiliary variables as shown in Methods Table 5. The imputation of register data was stratified by age to represent the use of parental values as a proxy for individuals aged below 30 years. As recommended (8,9), the outcome, censoring variables, and population weights were included as auxiliary variables. In Methods Table 6, the distribution of complete and imputed data is shown.

Methods Table 1: Overview of missing data in the cohort in four regions of Denmark, 2013 and 2017

	**All individuals N = 162,497**
**Overall**	
Missing on any variable, N (%)	20,856 (12.8)
Missing on survey data, N (%)	15,033 (9.3)
Missing on register data at baseline, N (%)	3,089 (1.9)
Missing on register data at index or pseudo-index date, N (%)	6,435 (4.0)
**Survey data**	
Missing on loneliness, N (%)	9,485 (5.8)
Missing on social isolation, N (%)	13,078 (8.0)
Missing on social support, N (%)	7,153 (4.4)
Missing on all survey variables above, N (%)	6,574 (4.0)
**Register data preceding survey participation**	
Missing on educational level, N (%)	3,089 (1.9)
Missing on annual disposable equivalised household income, N (%)	1,313 (0.8)
Missing on equivalised household wealth, N (%)	1,313 (0.8)
Missing on all register variables above, N (%)	1,313 (0.8)
**Register data preceding index or pseudo-index date**	
Missing on educational level, N (%)	6,433 (4.0)
Missing on annual disposable equivalised household income, N (%)	5,019 (3.1)
Missing on equivalised household wealth, N (%)	5,019 (3.1)
Missing on all register variables above, N (%)	5,017 (3.1)

Absolute numbers and proportions are unweighted.

Methods Table 2: Quantitative bias analysis among the 162,497 individuals with register linkage, using circulatory conditions as the outcome, in four regions of Denmark, 2013-2021

	Events/person-years at risk	IRR (95% CI)
**Disease-free individuals at baseline (N = 103,542)**		
Included in CCA	12,777/472,570	1 (ref.)
Missing survey data	1,335/38,691	1.22 (1.14–1.31)*
Missing register data	488/24,695	1.06 (0.95–1.19)*
**Disease-free individuals at baseline with complete survey data (N = 95,685)**		
Included in CCA	12,777/472,570	Ref.
Missing register data	422/21,591	1.05 (0.94–1.18)**
**Disease-free individuals at baseline with complete register data (N = 98,317)**		
Included in CCA	12,777/472,570	1 (ref.)
Missing survey data	1,269/35,586	1.17 (1.09–1.26)***

CCA: Complete case analysis; CI: Confidence interval; IRR: Incidence rate ratio. The number of events and person-years at risk are unweighted, whereas the IRR estimates are weighted based on register data to represent the population of the included regions in 2013 and 2017.
* Adjusted for age, sex, and year of survey participation
** Adjusted for age, sex, year of survey participation, loneliness, social isolation, and low social support
*** Adjusted for age, sex, year of survey participation, country of birth, educational level, income, and wealth preceding survey participation

Methods Table 3: Baseline characteristics of individuals with missing data in four regions of Denmark, 2013 and 2017

	Included in CCA (N = 141,641)	Missing survey data (N = 15,033)	Missing register data (N = 6,688)
Age, mean (SD)	48.3 (18.7)	53.3 (22.4)	40.5 (17.2)
Women, N (%)	76,126 (50.3)	8,339 (52.1)	3,626 (52.2)
Survey participation in 2013 as opposed to 2017, N (%)	28,732 (22.0)	3,525 (21.6)	1,173 (16.4)
Born abroad, N (%)	7,017 (7.0)	1,426 (15.6)	4,388 (77.3)
Living with a partner (self-reported), N (%)	99,227 (63.5)	4,308 (48.5)	4,249 (64.3)
Emotional limitations in daily activities (item 6–7 in SF-12), mean (SD)	8.6 (2.0)	7.9 (2.4)	8.2 (2.2)
The sum of the Perceived Stress Scale, mean (SD)	12.1 (7.3)	13.8 (7.4)	14.0 (7.1)
Enrolled in education c.f. register data, N (%)	11,207 (11.2)	1,093 (9.5)	597 (12.1)
In employment c.f. register data, N (%)	79,227 (55.7)	5,476 (37.8)	3,458 (52.8)
Cohabitation c.f. register data, N (%)	108,321 (71.1)	9,554 (58.4)	4,646 (65.3)

CCA: Complete case analysis; SF-12: 12-Item Short Form Survey. Absolute numbers are unweighted, whereas means, standard deviations, and percentages are weighted based on register data to represent the population of the included regions in 2013 and 2017.

Methods Table 4: Variables applied in imputation of survey data among individuals in four regions of Denmark, 2013 and 2017

	N (%) missing	Range	Mean (SD)
**Imputed variables**			
Three-Item Loneliness Scale, item 1	8,471 (5.2)	1 to 3	1.3 (0.5)
Three-Item Loneliness Scale, item 2	8,497 (5.2)	1 to 3	1.4 (0.6)
Three-Item Loneliness Scale, item 3	8,911 (5.5)	1 to 3	1.3 (0.5)
Social isolation, living alone	8,386 (5.2)	0 to 1	0.2 (0.4)
Social isolation, out of employment and not enrolled in education	9,269 (5.7)	0 to 1	0.4 (0.5)
Social isolation, less than monthly contact with friends	8,383 (5.2)	0 to 1	0.1 (0.3)
Social isolation, less than monthly contact with family	7,399 (4.6)	0 to 1	0.1 (0.3)
The social support item	7,153 (4.4)	1 to 4	1.6 (0.8)
**Analysis model variables**			
Population weights*	0 (0)	1 to 226	29.2 (19.6)
Age**	0 (0)	16 to 103	52.2 (18.4)
Female	0 (0)	0 to 1	0.5 (0.5)
Born abroad	≤5 (NA)	0 to 1	0.1 (0.3)
Year of survey	0 (0)	2,013 to 2,017	2,016.2 (1.6)
Mental disorder during follow-up	0 (0)	0 to 1	0.02 (0.1)
All-cause dementia during follow-up	0 (0)	0 to 1	0.01 (0.1)
Circulatory condition during follow-up	0 (0)	0 to 1	0.1 (0.3)
Endocrine condition during follow-up	0 (0)	0 to 1	0.05 (0.2)
Pulmonary condition or allergy during follow-up	0 (0)	0 to 1	0.1 (0.2)
Gastrointestinal condition during follow-up	0 (0)	0 to 1	0.04 (0.2)
Urogenital condition during follow-up	0 (0)	0 to 1	0.02 (0.2)
Musculoskeletal condition during follow-up	0 (0)	0 to 1	0.1 (0.3)
Hematologic condition during follow-up	0 (0)	0 to 1	0.02 (0.1)
Neurologic condition during follow-up	0 (0)	0 to 1	0.1 (0.3)
Cancer during follow-up	0 (0)	0 to 1	0.05 (0.2)
Death during follow-up	0 (0)	0 to 1	0.1 (0.2)
Emigration during follow-up	0 (0)	0 to 1	0.02 (0.1)
Prior mental disorder	0 (0)	0 to 1	0.1 (0.2)
**Auxiliary variables at survey participation**			
Prior psychopharmacological redemption	0 (0)	0 to 1	0.3 (0.4)
Prior consultation with private practising psychiatrist	0 (0)	0 to 1	0.1 (0.2)
Prior mental disorder, self-reported	11,684 (7.2)	0 to 1	0.1 (0.4)
Enrolled in education cf. register data	1,409 (0.9)	0 to 1	0.1 (0.3)
In employment cf. register data	1,409 (0.9)	0 to 1	0.5 (0.5)
Cohabitation cf. register data	1,409 (0.9)	0 to 1	0.8 (0.4)
Living with a partner, self-reported	9,240 (5.7)	0 to 1	0.7 (0.5)
Long-term disease, self-reported	7,691 (4.7)	0 to 1	0.4 (0.5)
The sum of the Perceived Stress Scale*	10,717 (6.6)	0 to 40	11.8 (7.1)
Score on getting enough sleep to feel rested	6,469 (4.0)	1 to 3	1.5 (0.7)
Spending time unwanted alone*	7,293 (4.5)	1 to 4	3.2 (0.9)
Evaluation of own health (item 1 in SF-12)*	1,081 (0.7)	1 to 5	2.6 (0.9)
Emotional limitations in daily activities (item 6–7 in SF-12)*	5,074 (3.1)	2 to 10	8.6 (2.0)
Mental health and vitality (item 9–11 in SF-12)*	6,132 (3.8)	3 to 15	9.3 (1.6)
Social contact limited due to physical or mental health (item 12 in SF-12)*	3,188 (2.0)	1 to 5	4.5 (0.9)

NA: Not applicable; SF-12: 12-Item Short Form Survey. Absolute numbers, percentages, means, and SDs are unweighted. The range is shown using means of the 5 lowest and highest values.

* Included in the imputation model as a linear term

** Included in the imputation model as a natural cubic spline with 3 knots

Methods Table 5: Variables applied in imputation of register data among individuals in four regions of Denmark, 2013 and 2017

	**Preceding survey participation**						**Preceding index or pseudo-index date**					
	**Aged 16-29 years (N = 24,953): Imputation of parental educational level, income, and wealth**			**Aged ≥30 years (N = 137,544): Imputation of own educational level, income, and wealth**			**Aged <30 years (N = 43,184): Imputation of parental educational level, income, and wealth**			**Aged ≥30 years (N = 119,313): Imputation of own educational level, income, and wealth**		
	**N (%) missing**	**Range**	**Mean (SD)**	**N (%) missing**	**Range**	**Mean (SD)**	**N (%) missing**	**Range**	**Mean (SD)**	**N (%) missing**	**Range**	**Mean (SD)**
**Imputed variables**												
Parental/own educational level	1,373 (5.5)	1 to 3	2.1 (0.7)	1,716 (1.2)	1 to 3	1.9 (0.7)	3,450 (8.0)	1 to 3	2.2 (0.7)	2,983 (2.5)	1 to 3	2.0 (0.7)
Parental/own annual disposable equivalised household income (1,000 DKK)	1,299 (5.2)	-11,201 to 11,696	319.6 (340.0)	14 (0.0001)	-9,683 to 14,935	293.9 (233.2)	3,313 (7.7)	-4,417 to 17,028	266.4 (295.5)	1,706 (1.4)	-3,690 to 19,480	263.9 (216.3)
Parental/own equivalised household wealth (1,000 DKK)	1,299 (5.2)	-68,863 to 134,658	479.8 (3081.8)	14 (0.0001)	-144,231 to 244,313	686.8 (3124.5)	3,313 (7.7)	-63,079 to 109,924	425.1 (2015.1)	1,706 (1.4)	-31,187 to 141,447	579.2 (2024.8)
Born abroad*	≤5 (NA)	0 to 1	0.1 (0.3)	≤5 (NA)	0 to 1	0.1 (0.3)	≤5 (NA)	0 to 1	0.1 (0.3)	≤5 (NA)	0 to 1	0.1 (0.2)
**Analysis model variables**												
Population weights**	0 (0)	2 to 222	42.2 (24.7)	0 (0)	1 to 217	26.8 (17.5)	0 (0)	2 to 226	39.5 (23.6)	0 (0)	1 to 203	25.5 (16.4)
Mean of parental/own age***	1,309 (5.2)	34 to 77	53.4 (5.8)	0 (0)	30 to 103	57.5 (14.5)	3,299 (7.6)	23 to 81	49.4 (7.2)	0 (0)	30 to 99	51.3 (12.9)
Female	NA	NA	NA	0 (0)	0 to 1	0.5 (0.5)	NA	NA	NA	0 (0)	0 to 1	0.5 (0.5)
Calendar year**	0 (0)	2,013 to 2,017	2016.2 (1.6)	0 (0)	2,013 to 2,017	2,016.2 (1.6)	0 (0)	1,995 to 2,017	2006.9 (5.5)	0 (0)	1,995 to 2,017	2006.5 (5.7)
Mental disorder during follow-up	0 (0)	0 to 1	0.04 (0.2)	0 (0)	0 to 1	0.01 (0.1)	0 (0)	0 to 1	0.03 (0.2)	0 (0)	0 to 1	0.01 (0.1)
All-cause dementia during follow-up	NA	NA	NA	0 (0)	0 to 1	0.01 (0.1)	NA	NA	NA	0 (0)	0 to 1	0.01 (0.1)
Circulatory condition during follow-up	0 (0)	0 to 1	0.02 (0.1)	0 (0)	0 to 1	0.1 (0.3)	0 (0)	0 to 1	0.04 (0.2)	0 (0)	0 to 1	0.1 (0.3)
Endocrine condition during follow-up	0 (0)	0 to 1	0.02 (0.1)	0 (0)	0 to 1	0.1 (0.2)	0 (0)	0 to 1	0.03 (0.2)	0 (0)	0 to 1	0.1 (0.2)
Pulmonary condition or allergy during follow-up	0 (0)	0 to 1	0.1 (0.2)	0 (0)	0 to 1	0.1 (0.3)	0 (0)	0 to 1	0.1 (0.2)	0 (0)	0 to 1	0.1 (0.3)
Gastrointestinal condition during follow-up	0 (0)	0 to 1	0.01 (0.1)	0 (0)	0 to 1	0.04 (0.2)	0 (0)	0 to 1	0.01 (0.1)	0 (0)	0 to 1	0.05 (0.2)
Urogenital condition during follow-up	0 (0)	0 to 1	0.002 (0.04)	0 (0)	0 to 1	0.03 (0.2)	0 (0)	0 to 1	0.002 (0.04)	0 (0)	0 to 1	0.03 (0.2)
Musculoskeletal condition during follow-up	0 (0)	0 to 1	0.1 (0.2)	0 (0)	0 to 1	0.1 (0.3)	0 (0)	0 to 1	0.1 (0.3)	0 (0)	0 to 1	0.1 (0.3)
Hematologic condition during follow-up	0 (0)	0 to 1	0.003 (0.1)	0 (0)	0 to 1	0.02 (0.1)	0 (0)	0 to 1	0.004 (0.1)	0 (0)	0 to 1	0.02 (0.1)
Neurologic condition during follow-up	0 (0)	0 to 1	0.03 (0.2)	0 (0)	0 to 1	0.1 (0.3)	0 (0)	0 to 1	0.03 (0.2)	0 (0)	0 to 1	0.1 (0.3)
Cancer during follow-up	0 (0)	0 to 1	0.004 (0.1)	0 (0)	0 to 1	0.1 (0.2)	0 (0)	0 to 1	0.01 (0.1)	0 (0)	0 to 1	0.1 (0.2)
Death during follow-up	0 (0)	0 to 1	0.002 (0.04)	0 (0)	0 to 1	0.1 (0.2)	0 (0)	0 to 1	0.002 (0.05)	0 (0)	0 to 1	0.1 (0.3)
Emigration during follow-up	0 (0)	0 to 1	0.1 (0.2)	0 (0)	0 to 1	0.01 (0.1)	0 (0)	0 to 1	0.05 (0.2)	0 (0)	0 to 1	0.01 (0.1)
Pre-existing mental disorder	0 (0)	0 to 1	0.1 (0.3)	0 (0)	0 to 1	0.1 (0.2)	0 (0)	0 to 1	0.1 (0.3)	0 (0)	0 to 1	0.1 (0.2)
**Auxiliary variables**												
Mother's age at childbirth***	1,714 (6.9)	15 to 47	29.3 (4.7)	NA	NA	NA	4,154 (9.6)	14 to 47	28.4 (4.9)	NA	NA	NA
Number of children in own/parents' household(s)**	1,299 (5.2)	0 to 7	0.2 (0.5)	14 (0.0001)	0 to 8	0.4 (0.8)	3,312 (7.7)	0 to 8	0.7 (1.0)	1,705 (1.4)	0 to 7	0.6 (0.9)
Number of adults in own/parents' household(s)**	1,299 (5.2)	1 to 7	2.6 (0.9)	14 (0.0001)	1 to 7	1.9 (0.7)	3,312 (7.7)	1 to 7	2.4 (0.9)	1,705 (1.4)	1 to 6	2.0 (0.7)
Parental/own educational level 5 years prior	1,452 (5.8)	1 to 3	2.1 (0.7)	3,348 (2.4)	1 to 3	1.9 (0.7)	3,534 (8.2)	1 to 3	2.2 (0.7)	3,772 (3.2)	1 to 3	2.0 (0.7)

	**Preceding survey participation Aged 16-29 years (N = 24,953): Imputation of parental educational level, income, and wealth**			**Aged ≥30 years (N = 137,544): Imputation of own educational level, income, and wealth**			**Preceding index or pseudo-index date Aged <30 years (N = 43,184): Imputation of parental educational level, income, and wealth**			**Aged ≥30 years (N = 119,313): Imputation of own educational level, income, and wealth**		
	**N (%) missing**	**Range**	**Mean (SD)**	**N (%) missing**	**Range**	**Mean (SD)**	**N (%) missing**	**Range**	**Mean (SD)**	**N (%) missing**	**Range**	**Mean (SD)**
Parental/own annual disposable equivalised household income 5 years prior (1,000 DKK)**	1,377 (5.5)	-8,467 to 5,927	274.8 (238.6)	1,707 (1.2)	-13,509 to 20,609	273.5 (251.4)	3,419 (7.9)	-3,597 to 8,074	236.3 (166.7)	2,597 (2.2)	-5,125 to 10,469	244.9 (165.3)
Parental/own equivalised household wealth 5 years prior (1,000 DKK)**	1,377 (5.5)	-68,505 to 120,113	424.1 (2896.4)	1,707 (1.2)	-47,956 to 153,617	664.4 (2185.5)	3,419 (7.9)	-65,908 to 96,464	260.7 (1,999.8)	2,597 (2.2)	-29,296 to 105,270	408.0 (1564.5)
Offspring's annual disposable equivalised household income (1,000 DKK)**	≤5 (NA)	-2,061 to 9,925	217.0 (230.1)	NA	NA	NA	2,730 (6.3)	-2,350 to 4,623	205.6 (126.7)	NA	NA	NA
Offspring's equivalised household wealth (1,000 DKK)**	≤5 (NA)	-11,378 to 95,531	157.2 (1,991.4)	NA	NA	NA	2,825 (6.5)	-24,994 to 72,034	167.2 (1,266.2)	NA	NA	NA

NA: Not applicable. Absolute numbers, percentages, means, and SDs are unweighted. The range is shown using means of the 5 lowest and highest values.

* We included the imputed data on this variable from the imputation model using data preceding survey participation

** Included in the imputation model as a linear term

*** Included in the imputation model as a natural cubic spline with 3 knots

Methods Table 6: Distribution of variables in complete and imputed data among individuals in four regions of Denmark, 2013 and 2017

	**Complete data**	**Imputed data**
**Survey data**		
Lonely, N (%)	8,937 (7.3)	871 (11.0)
Socially isolated, N (%)	3,963 (3.2)	753 (6.6)
Low social support, N (%)	20,264 (14.7)	1,096 (17.0)
**Register data preceding survey participation**		
Educational level		
Lowest (ISCED 0-2), N (%)	36,523 (25.5)	929 (29.9)
Middle (ISCED 3-5), N (%)	77,979 (47.6)	1,477 (48.2)
Highest (ISCED 6-8), N (%)	44,906 (26.9)	683 (21.9)
Annual disposable equivalised household income (1,000 DKK), mean (SD)	286 (251)	271 (311)
Equivalized household wealth (1,000 DKK), mean (SD)	552 (3,069)	387 (2,300)
**Register data preceding index or pseudo-index date**		
Educational level		
Lowest (ISCED 0-2), N (%)	43,327 (31.0)	2,092 (34.0)
Middle (ISCED 3-5), N (%)	75,977 (47.5)	2,976 (46.2)
Highest (ISCED 6-8), N (%)	36,760 (21.6)	1,365 (19.8)
Annual disposable equivalised household income (1,000 DKK), mean (SD)	255 (228)	216 (424)
Equivalized household wealth (1,000 DKK), mean (SD)	476 (2,038)	255 (2,824)

Absolute numbers are unweighted, whereas means, SDs, and proportions are weighted based on register data to represent the population of the included regions in 2013 and 2017.

Methods S3. Main analysis

Adjustments

Age was included as a time-varying covariate in 1-year incremental age groups and modelled as a natural cubic spline with 5 knots. For analyses in subgroups based on age group (16–65 and >65 years), we remodelled age using a natural cubic spline with 4 knots fitted for the specific age group. Income and wealth were modelled as categorical variables using quartiles based on the distribution of values in the study population.

Sensitivity analyses on potential reverse causation

As a sensitivity analysis, we repeated the main analysis with:

i) Exclusion of individuals with a preceding or current self-reported medical conditions within the investigated category and a delayed start of follow-up to 6 months after baseline to reduce the likelihood of including individuals with an undiagnosed medical condition. For this analysis, we applied self-reported information from the Danish National Health Survey on circulatory conditions (hypertension, angina pectoris, acute myocardial infarction, and stroke), endocrine conditions (diabetes mellitus), pulmonary conditions (chronic pulmonary disease, asthma, and allergy), musculoskeletal conditions (rheumatoid arthritis and osteoporosis), neurologic conditions (cataract, tinnitus, and migraine or frequent headache), and cancer (unspecified cancer).

ii) Adjustment for self-rated general health in the Danish National Health Survey (item 1 from the 12-Item Short Form Survey), thus assuming that health status at baseline acts purely as a confounder. Self-rate general health was included as a categorical variable with each five response options constituting a category.

Methods S4. Interaction analysis

Pseudo-index dates

To assign pseudo-index dates to individuals without a pre-existing mental disorder, we replicated the distribution of time since diagnosis among individuals with a mental disorder. After stratification by sex and age group at baseline (16-25, >25-35, >35-45, >45-55 >55-65, and >65 years), we assigned random numbers (0;1) to individuals without a pre-existing mental disorder to simulate the cumulate distribution among individuals with a pre-existing mental disorder. Furthermore, new random numbers were drawn for 38 individuals (0.02%) who were first given a pseudo-index date at a lower age than any of the individuals with a pre-existing mental disorder.

Adjustment procedures

Like the main analysis, age was included as a time-varying covariate in 1-year incremental age groups and modelled as a natural cubic spline with 5 knots. Income and wealth were modelled as categorical variables using quartiles based on the distribution of values in the study population.

Relative Excess Risk due to Interaction

The Relative Excess Risk due to Interaction (RERI) is calculated as the relative risk for each of the singly exposed groups (RR_{01} and RR_{10}) subtracted from the relative risk for the doubly exposed group (RR_{11}) and added with 1 to only subtract the reference risk once (10):

$$RERI = RR_{11} - RR_{01} - RR_{10} + 1$$

Sensitivity analyses on the operationalization of pre-existing mental disorders

As a sensitivity analysis on the operationalization of mental disorders, we repeated the main analysis with a broader definition of mental disorders additionally including the following indicators:

i) Redeemed prescription for psychopharmacological treatment in 18 years preceding survey participation recorded in the Danish National Prescription Registry including antipsychotics (N05A except N05AN), antidepressants (N06A), lithium (N05AN), anxiolytics (N05B except N05BA01), medication for ADHD (C02AC02, N06BA02, N06BA04, N06BA09, and N06BA12), and medication for alcohol and opioid dependence (N07BB-N07BB04), based on a recent study (11)

ii) Consultation with a private practicing psychiatrist in 18 years preceding survey participation recorded in the Danish National Health Service Register (12)

iii) Preceding or current mental disorder self-reported in the Danish National Health Survey

Supplementary Figures

Fig. S1. Flowchart depicting the initial study population in four regions of Denmark, 2013 and 2017

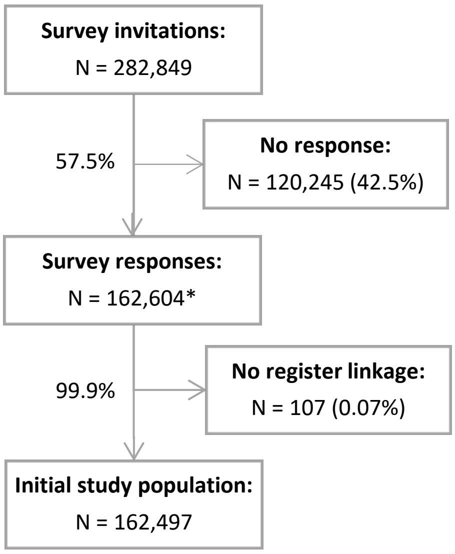

* For a small number (N = 23) of survey participants in 2013, the specific date of survey participation was missing and replaced with the median date.

Fig. S2. Venn diagrams of the distribution of loneliness, social isolation, and low social support among women and men in four regions of Denmark, 2013 and 2017

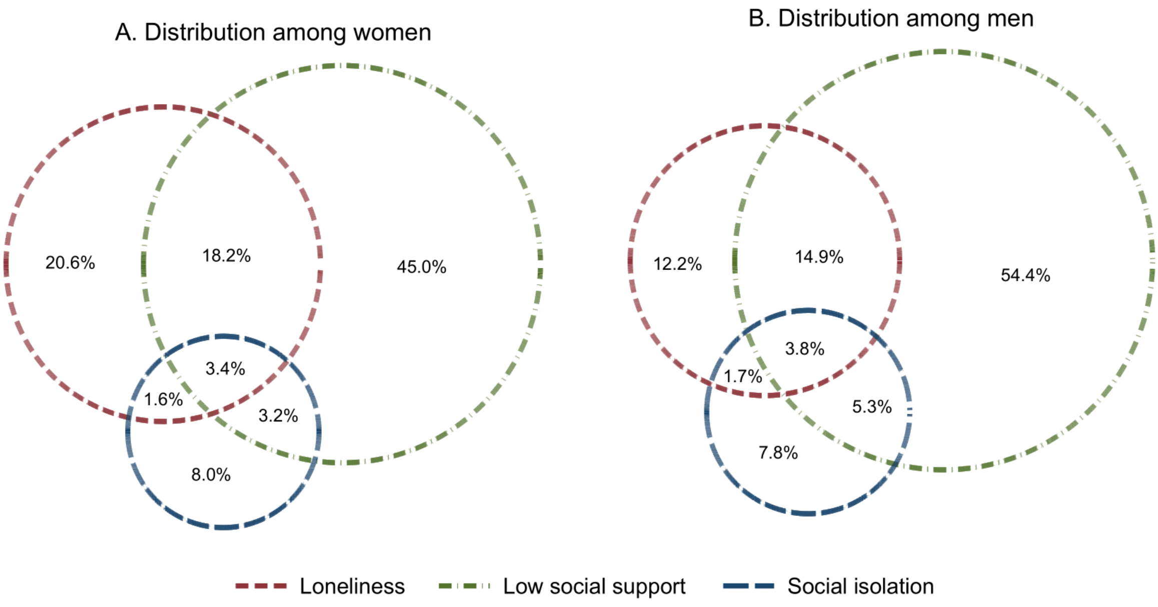

Missing data were imputed using multiple imputation by chained equations. Proportions are shown for individuals with at least one of the indicators of social disconnection and weighted based on register data to represent the population of the included regions in 2013 and 2017.

Fig. S3. Sensitivity analysis of social disconnectedness and rates of subsequent medical conditions in 11 broad categories in four regions of Denmark, 2013-2021

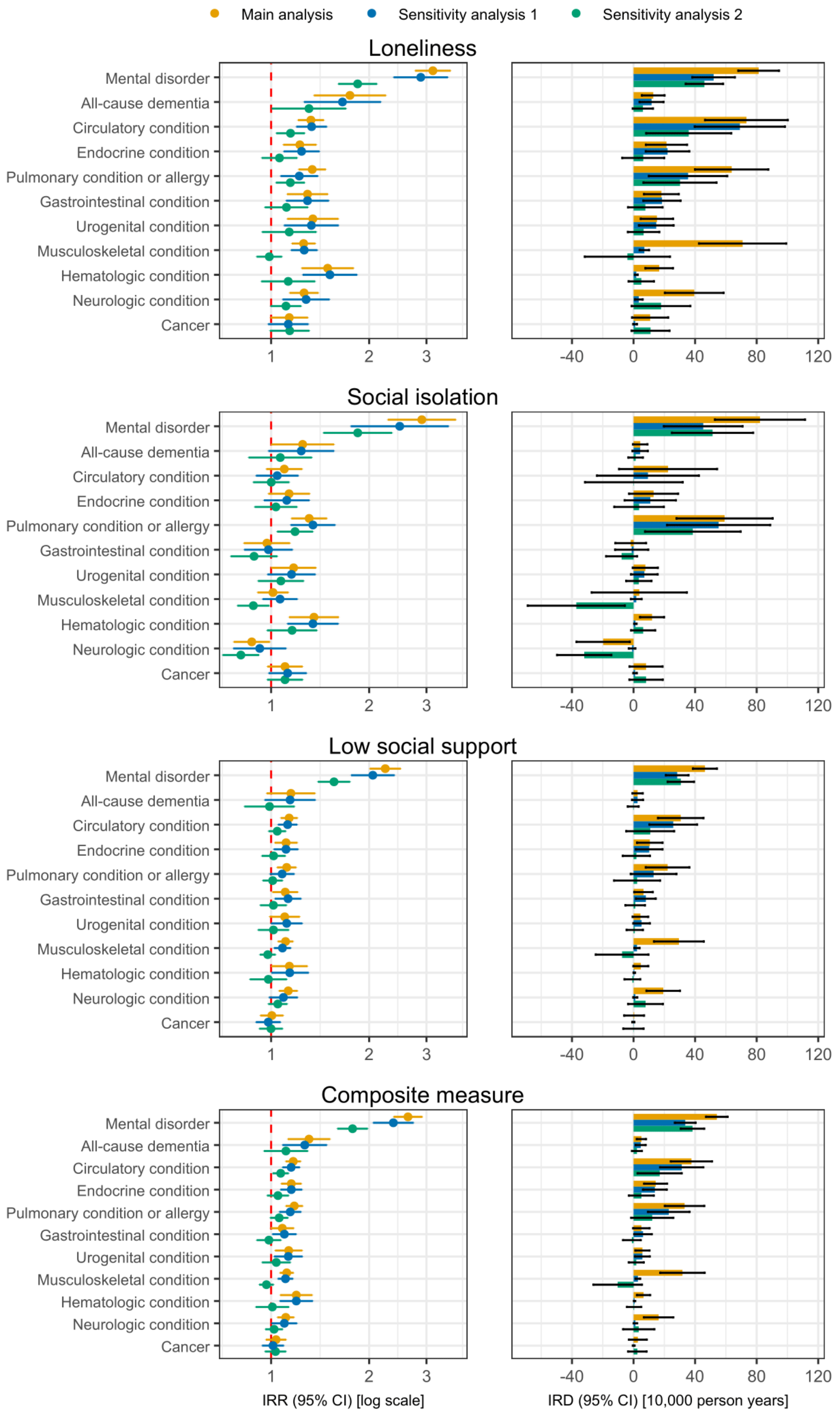

CI: Confidence interval; IRD: Incidence rate difference; IRR: Incidence rate ratio. IRRs are displayed on a logarithmic scale, whereas IRDs are displayed on a linear scale. Missing data was imputed using multiple imputation by chained equations, and the results are weighted based on register data to represent the population of the included regions in 2013 and 2017. In this sensitivity analysis, we excluded individuals with self-reported disease, delayed the start of follow-up by 6 months, and adjusted for self-rated general health. IRDs are calculated using marginal standardization to individuals with versus without at least one of loneliness, social isolation, and low social support. All estimates are additionally adjusted for age, sex, year of survey participation, country of birth, educational level, income, and wealth (Model 2).

Fig. S4. Interaction between social disconnectedness and pre-existing mental disorders on sex-specific rates of subsequent medical conditions in 10 broad categories in four regions of Denmark, 2013-2021

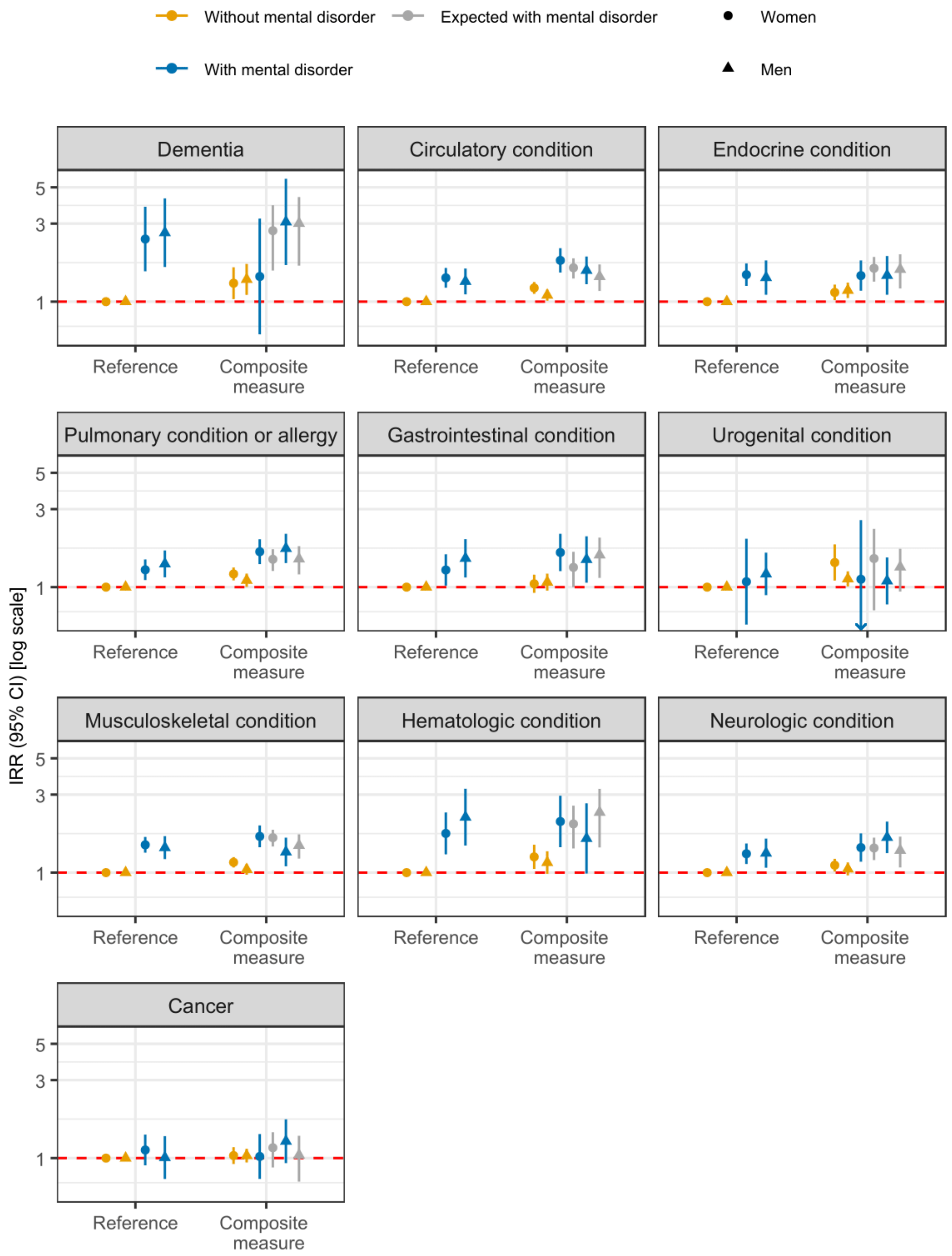

CI: Confidence interval; IRR: Incidence rate ratio; RERI: Relative Excess Risk due to Interaction. IRRs are displayed on a logarithmic scale. Missing data was imputed using multiple imputation by chained equations, and the results are weighted based on register data to represent the population of the included regions in 2013 and 2017. The estimates of expected with a mental disorder are calculated based on additive interaction between mental disorders and the composite measure (at least one of loneliness, social isolation, and low social support). All estimates are adjusted for age, year of survey participation, country of birth, educational level, income, and wealth (Model 2).

Fig. S5. Sensitivity analysis on interaction between social disconnectedness and pre-existing mental disorders on rates of subsequent medical conditions in 10 broad categories in four regions of Denmark, 2013-2021

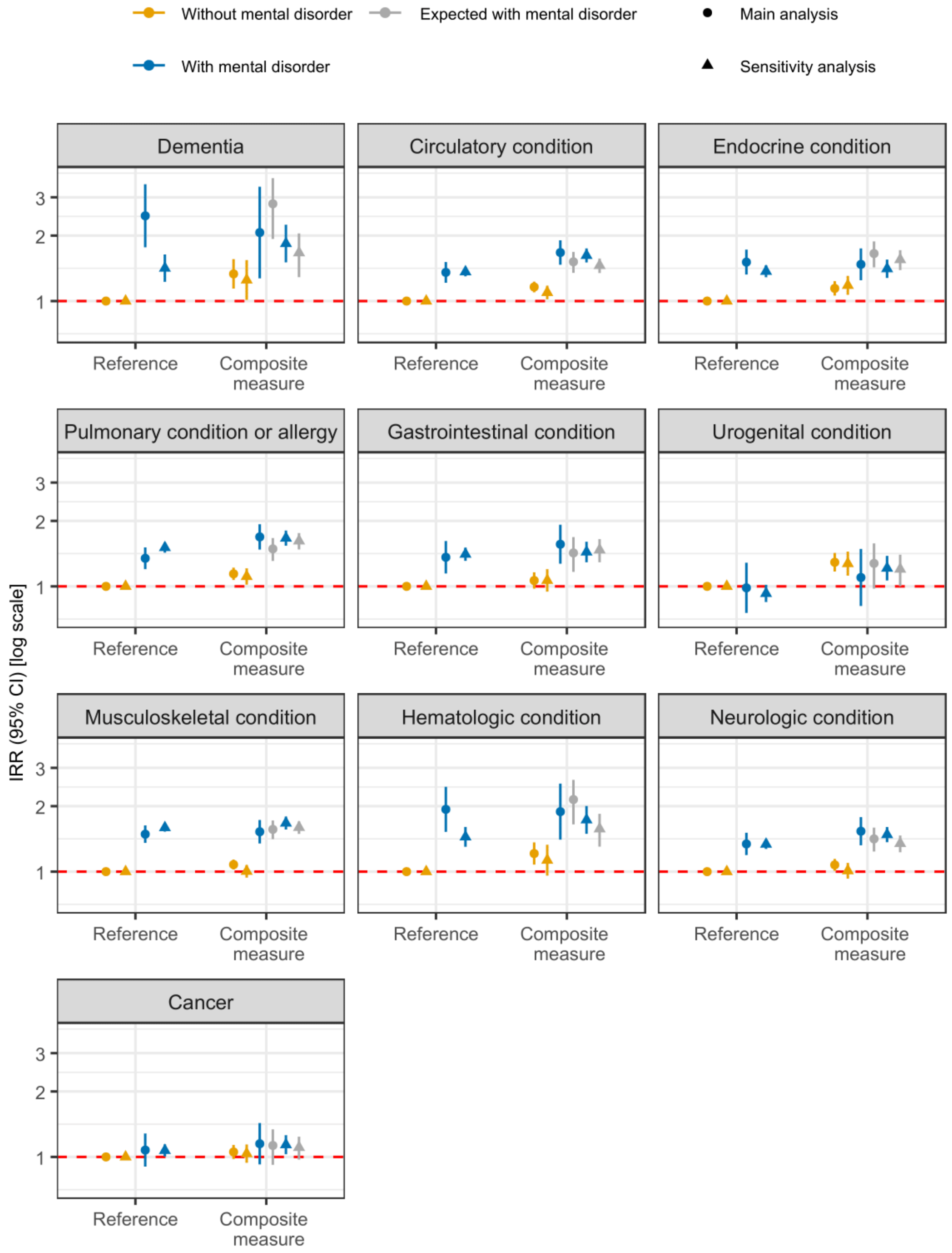

CI: Confidence interval; IRR: Incidence rate ratio. IRRs are displayed on a logarithmic scale. Missing data was imputed using multiple imputation by chained equations, and the results are weighted based on register data to represent the population of the included regions in 2013 and 2017. In this sensitivity analysis, we used a broader definition of mental disorders additionally including self-reported information, redeemed prescriptions of psychopharmaceuticals, and consultations with private practicing psychiatrists. The estimates of expected with a mental disorder are calculated based on additive interaction between mental disorders and the composite measure (at least one of loneliness, social isolation, and low social support). All estimates are adjusted for age, sex, year of survey participation, country of birth, educational level, income, and wealth (Model 2).

Supplementary Tables

Table S1: Overview of systematic reviews and meta-analyses of longitudinal studies assessing social disconnectedness and subsequent medical conditions

Author and publication year	Medical condition	Search strategy	Specific inclusion criteria	Measures of social disconnectedness, number of studies, and accumulated study population	Results
Gariépy et al. 2016 (13)	Depression	3 databases searched until February 2015	• Only Western countries, including the USA, Canada and Europe (EU and member states of the European Free Trade Association), Australia, and New Zealand	Social support: 31 studies (N = 58,883)	No pooled estimate provided for longitudinal studies separately, except that 26 studies (84%) provided a significant result
Kuiper et al. 2015 (14)	Dementia	3 databases searched until July 2012	• None	Loneliness: 4 studies (N = 4,758)<br>Social isolation*: 9 studies (N = 17,528)	Higher pooled risk among individuals with less frequent social contact (relative risk 1.57 [95% CI 1.32 to 1.85] based on 8 of the studies) and individuals with loneliness (relative risk 1.58 [95% CI 1.19 to 2.09] based on 3 of the studies)
Lara et al. 2019 (15)	Dementia	6 databases searched until November 2018	• Studies including middle- and older-aged individuals (≥50 years) from the general community-based population<br>• No cognitive impairment at baseline<br>• Studies must have lasted at least one year	Loneliness: 10 studies (N = 37,339)	Pooled risk ratio 1.26 (95% CI 1.14 to 1.40) based on 8 of the studies
Mann et al. 2022 (16)	Mental disorders	6 databases searched until August 2021	• Included individuals aged ≥16 years<br>• Did not include studies on intellectual disabilities or organic mental disorders<br>• Did not include cohorts selected on the basis of a primary physical health diagnosis	Loneliness: 32 studies (N = 651,217)	Based on 7 seven studies with odds ratios, a pooled odds ratio of 2.33 (95% CI 1.62 to 3.34) for risk of new onset depression in adults who were often lonely
Mansfield et al. 2018 (17)	Carpal tunnel syndrome	3 databases searched until May 2017	• Included adult individuals (>18 years)<br>• Only clinically diagnosed carpal tunnel syndrome<br>• Must have investigated a minimum of 1 or more psychosocial factors	Social support: 2 studies (N = 9,173)	Higher risk of onset for low social support in one study (odds ratio 1.2 [95% CI, 0.90-1.80]), but lower risk of onset for high social support in the other study (hazard ratio 0.54 [95% CI, 0.31-0.95])
Penninkilampi et al. 2018 (18)	Dementia	8 databases searched between January 2012 to May 2017.	• None	Loneliness: 4 studies (N = 4,698)<br>Social isolation*: 3 studies (N not provided)<br>Social support: 5 studies (N not provided)	Higher pooled risk for loneliness (relative risk 1.38 [95% CI 0.98 to 1.94]), social isolation (relative risk 1.54 [95% CI 1.14 to 2.09]) and poor social support (relative risk 1.28 [95% CI 1.01 to 1.62])
Santini et al. 2015 (19)	Depression	3 databases searched between 2004 and 2014	• Studies including a sample that aimed to reflect the general population	Social support: 23 studies** (N = 77,635)	No pooled estimate provided
Schwarzbach et al. 2014	Depression	6 databases searched between January 2000 and December 2012	• Studies including persons aged ≥60 years from nationally or regionally representative studies<br>• Studies with a multivariate analysis adjusting for confounders	Social isolation: 3 (N = 4,943**)<br>Social support: 4 (N = 10,376)	No pooled estimate provided, except that 3 studies (75%) provided a significant result for social support and 1 study (33%) for social isolation (frequency of contacts)
Valtorta et al. 2016 (20)	Coronary heart disease and stroke	16 databases searched until May 2015	• Only studies conducted in high-income countries<br>• Coronary heart disease or stroke must be the first instance of diagnosis, except where analyses controlled for previous events	Loneliness or social isolation: 23 studies (N = 181,006)	Pooled relative risk of incident coronary heart disease and stroke was 1.29 (95% CI 1.04 to 1.59) and 1.32 (95% CI 1.04 to 1.68) respectively

Wang et al. 2023 (21)	Dementia	5 databases searched until August 2021	• Studies including middle-aged or older adults (>50 years) who are cognitively intact at baseline<br>• No other diseases that confound the risk of dementia	Loneliness: 10 studies (N = 62,644**)<br>Social isolation: 5 studies (N = 49,727)<br>Social support: 8 studies (N = 49,008**)	Higher pooled risk for loneliness (relative risk 1.42 [95% CI 1.26 to 1.60]) and social isolation (relative risk 1.58 [95% CI 0.80 to 3.12]) and lower pooled risk for high social support (relative risk 0.92 [95% CI 0.80 to 1.06]) based on 7 studies
Yang et al. 2023 (22)	Sarcopenia	4 databases searched until June 2023	• Must describe the diagnostic criteria of sarcopenia in detail<br>• Must report odds ratio	Social isolation: 2 studies (N = 5,611)	Pooled odds ratio 1.79 (95% CI 1.55 to 2.06)

Based on a literature search in the NCBI PubMed Database on the 20th of December 2023 with inclusion of systematic reviews of longitudinal studies excluding reviews of studies from one country or patient group and reviews on individuals at a specific time in the life (e.g., postpartum women).

* Mentioned as frequency of social contact or few social contacts

** Not reported for one of the studies

Table S2. List of diagnosis and prescription codes in each medical condition category

Medical condition category	Coding definition	Diagnosis/mortality codes (ICD-10)	Medication	
			Drug codes (ATC)	Time frame for prescriptions
**MENTAL DISORDER**				
Mental disorder	Diagnosis	F10-69, F80-99		
**ALL-CAUSE DEMENTIA**				
Alzheimer's disease	Diagnosis	F00, G30		
Vascular dementia	Diagnosis	F01		
Other dementia	Diagnosis	F02, F03, F1X.73, G23.1, G31.0, G31.1, G31.8B, G31.8E, G31.85		
**CIRCULATORY CONDITION**				
Hypertension	Diagnosis AND/OR prescriptions of antihypertensives [a]	I10-I13, I15	C02, C03, C04, C07, C08, C09	Twice in 1 year
Dyslipidaemia	Diagnosis AND/OR prescription of lipid-lowering drugs [b]	E78	C10	Twice in 1 year
Ischemic heart disease	Diagnosis AND/OR prescription for antianginal drug	I20-I25	C01DA	Twice in 1 year
Atrial fibrillation	Diagnosis	I48		
Heart failure	Diagnosis	I50		
Peripheral artery occlusive disease	Diagnosis	I70-I74		
Stroke	Diagnosis	I60-I64, I69		
**ENDOCRINE CONDITION**				
Diabetes mellitus	Diagnosis AND/OR prescription of antidiabetics	E10-E14	A10A, A10B	Twice in 1 year
Thyroid disorder	Diagnosis AND/OR prescription of thyroid therapy drugs	E00-E05, E061-E069, E07	H03	Twice in 1 year
Gout	Diagnosis	E79, M10		
**PULMONARY CONDITION OR ALLERGY**				
Chronic pulmonary disease	Diagnosis AND/OR Prescription for obstructive airway disease drugs	J40-J47 [c]	R03	Twice in 1 year
Allergy	Diagnosis AND/OR Prescription for non-sedative antihistamines AND/OR nasal antiallergics	J30.1-J30.4, L23, L50.0, T78.0, T78.2, T78.4 [c]	R06AX, R06AE07, R06AE09, R01AC, R01AD	Twice in 1 year
**GASTROINTESTINAL CONDITION**				
Ulcer/chronic gastritis	Diagnosis	K221, K25-K28, K293-K295		
Chronic liver disease	Diagnosis	B16-B19, K70-K74, K766, I85		
Inflammatory bowel disease	Diagnosis	K50-K51		
Diverticular disease of intestine	Diagnosis	K57		
**UROGENITAL CONDITION**				
Chronic kidney disease	Diagnosis	N03, N11, N18-N19		

Medical condition category	Coding definition	Diagnosis/mortality codes (ICD-10)	Medication	
			Drug codes (ATC)	Time frame for prescriptions
Prostate disorders	Diagnosis AND/OR prescription of prostate hyperplasia therapy drugs	N40	C02CA, G04C	Twice in 1 year
**MUSCULOSKELETAL CONDITION**				
Connective tissue disorders	Diagnosis	M05-M06, M08-M09, M30-M36, D86		
Osteoporosis	Diagnosis AND/OR prescription for osteoporosis drugs	M80-M82	M05B, G03XC01, H05AA	Twice in 1 year
Painful condition	Repeated prescriptions of analgesics		N02A, N02BA51, N02BE, M01A, M02A	4x in 1 year
**HEMATOLOGIC CONDITION**				
HIV/AIDS	Diagnosis	B20-B24		
Anaemias	Diagnosis	D50-D53, D55-D59, D60-D61, D63-D64		
**NEUROLOGIC CONDITION**				
Vision problem	Diagnosis	H25, H40, H54		
Hearing problem	Diagnosis	H90-H91, H931		
Migraine	Diagnosis AND/OR prescription of specific anti-migraine drugs	G43	N02C	Twice in 1 year
Epilepsy	Diagnosis AND prescription of anti-epileptics	G40-G41 [c]	N03	Twice in 1 year
Parkinson's disease	Diagnosis	G20-G22		
Multiple sclerosis	Diagnosis	G35		
Neuropathies	Diagnosis	G50-G64		
**CANCER**				
Cancer	Diagnosis	C00-C43, C45-C97		

ATC: Anatomical Therapeutic Chemical Classification System; ICD-10: International Statistical Classification of Diseases and Related Health Problems 10th Revision.

[a] Ascertained only in absence of ischemic heart disease/heart failure, and by diuretics only if no kidney disease

[b] Prescriptions used if no previous ischemic heart disease

[c] Amended in our application

Table S3. Additional characteristics of the cohort in four regions of Denmark, 2013 and 2017

	**Total sample: N = 162,497**	**Pre-existing mental disorder: N = 10,347 (7.7%)**	**No pre-existing mental disorder: N = 152,150 (92.3%)**
**At survey participation**			
Self-reported mental disorder, N (%)	24,406 (16.5)	7,266 (71.4)	17,141 (11.9)
Psychopharmacological redemption, N (%)	43,929 (26.2)	8,408 (80.3)	35,521 (21.7)
Consultation with a private practicing psychiatrist, N (%)	8,651 (5.8)	3,387 (32.2)	5,264 (3.6)
Self-reported circulatory condition, N (%)	47,889 (25.6)	2,721 (23.9)	45,168 (25.7)
Self-reported endocrine condition, N (%)	10,219 (5.7)	768 (7.0)	9,451 (5.6)
Self-reported pulmonary condition, N (%)	54,933 (34.2)	4,331 (42.1)	50,602 (33.6)
Self-reported musculoskeletal condition, N (%)	16,123 (9.2)	1,139 (10.7)	14,984 (9.1)
Self-reported neurologic condition, N (%)	64,453 (37.8)	4,956 (46.3)	59,497 (37.1)
Self-reported cancer, N (%)	11,713 (6.0)	557 (4.5)	11,156 (6.1)
Years since the index or pseudo-index date, mean (SD)	8.9 (6.5)	8.6 (6.9)	9.0 (6.4)
Increase in annual disposable equivalised household income since the index or pseudo-index date (1,000 DKK), mean (SD)	32.9 (380.7)	18.9 (201.7)	34.1 (390.8)
Increase in equivalised household wealth since the index or pseudo-index date (1,000 DKK), mean (SD)	85.8 (2,611.5)	-27.2 (1,028.2)	95.3 (2,712.6)
**At index or pseudo-index date**			
Educational level			
Lowest (ISCED 0-2), N (%)	45,419 (31.2)	3,613 (38.4)	41,806 (30.6)
Middle (ISCED 3-5), N (%)	78,953 (47.4)	4,752 (44.4)	74,201 (47.6)
Highest (ISCED 6-8), N (%)	38,125 (21.4)	1,982 (17.2)	36,143 (21.8)
Annual disposable equivalised household income (1,000 DKK), mean (SD)	253.1 (278.9)	222.3 (145.3)	255.6 (286.4)
Equivalised household wealth (1,000 DKK), mean (SD)	462.8 (2,468.3)	204.2 (1,017.2)	484.4 (2,557.0)

Missing data was imputed using multiple imputation by chained equations, and percentages are weighted based on register data to represent the population of the included regions in 2013 and 2017.

Table S4. Persons at risk at start of follow-up and cases during follow-up in the total cohort of 162,497 individuals in four regions of Denmark, 2013-2021

Medical condition category	Pre-existing cases before follow-up, N (%)	Persons at risk at start of follow-up, N (%)	Cases during follow-up/person-years at risk
**Total**			
Mental disorder	10,347 (6.4)	152,150 (93.6)	2,712/823,691*
All-cause dementia	627 (0.4)	161,870 (99.6)	1,438/881,315
Circulatory condition	58,955 (36.3)	103,542 (63.7)	14,534/532,851
Endocrine condition	19,807 (12.2)	142,690 (87.8)	7,511/762,665
Pulmonary condition or allergy	51,926 (32.0)	110,571 (68.0)	10,766/575,370
Gastrointestinal condition	9,175 (5.6)	153,322 (94.4)	5,938/823,719
Urogenital condition	7,478 (4.6)	155,019 (95.4)	4,034/838,629
Musculoskeletal condition	51,371 (31.6)	111,126 (68.4)	17,818/563,279
Hematologic condition	3,152 (1.9)	159,345 (98.1)	2,801/865,514
Neurologic condition	33,135 (20.4)	129,362 (79.6)	11,878/678,642
Cancer	10,288 (6.3)	152,209 (93.7)	7,523/819,347
**Women**			
Mental disorder	6,465 (7.4)	81,162 (92.6)	1,656/439,845*
All-cause dementia	328 (0.4)	87,299 (99.6)	757/476,596
Circulatory condition	30,975 (35.3)	56,652 (64.7)	7,185/293,017
Endocrine condition	12,214 (13.9)	75,413 (86.1)	4,217/402,721
Pulmonary condition or allergy	30,308 (34.6)	57,319 (65.4)	5,718/298,019
Gastrointestinal condition	4,843 (5.5)	82,784 (94.5)	3,059/446,022
Urogenital condition	585 (0.7)	87,042 (99.3)	527/475,404
Musculoskeletal condition	30,859 (35.2)	56,768 (64.8)	9,745/285,795
Hematologic condition	1,879 (2.1)	85,748 (97.9)	1,405/466,570
Neurologic condition	19,942 (22.8)	67,685 (77.2)	6,315/354,885
Cancer	5,551 (6.3)	82,076 (93.7)	3,547/443,430
**Men**			
Mental disorder	3,882 (5.2)	70,988 (94.8)	1,056/383,846*
All-cause dementia	299 (0.4)	74,571 (99.6)	681/404,718
Circulatory condition	27,980 (37.4)	46,890 (62.6)	7,349/239,834
Endocrine condition	7,593 (10.1)	67,277 (89.9)	3,294/359,944
Pulmonary condition or allergy	21,618 (28.9)	53,252 (71.1)	5,048/277,351
Gastrointestinal condition	4,332 (5.8)	70,538 (94.2)	2,879/377,697
Urogenital condition	6,893 (9.2)	67,977 (90.8)	3,507/363,225
Musculoskeletal condition	20,512 (27.4)	54,358 (72.6)	8,073/277,483
Hematologic condition	1,273 (1.7)	73,597 (98.3)	1,396/398,945
Neurologic condition	13,193 (17.6)	61,677 (82.4)	5,563/323,756
Cancer	4,737 (6.3)	70,133 (93.7)	3,976/375,917

Absolute numbers are unweighted, whereas percentages are weighted based on register data to represent the population of the included regions in 2013 and 2017.
*Due to data protection rules, a small number of individuals (≤5) who had the outcome on day 0 are included as cases here, but excluded in the final analyses

Table S5. Social disconnectedness and rates of subsequent medical conditions in 11 broad categories in four regions of Denmark, 2013-2021

		Model 1		Model 2	
	Events/person-years at risk among exposed	IRR (95% CI)	IRD per 10,000 person-years (95% CI)	IRR (95% CI)	IRD per 10,000 person-years (95% CI)
**Loneliness**					
Mental disorder	498/36,104	3.58 (3.16–4.05)	88 (74–101)	3.14 (2.77–3.56)	81 (68–95)
All-cause dementia	97/48,949	1.83 (1.42–2.35)	14 (6–21)	1.75 (1.35–2.25)	13 (5–20)
Circulatory condition	786/31,148	1.43 (1.31–1.56)	92 (65–119)	1.32 (1.21–1.45)	73 (46–100)
Endocrine condition	484/41,478	1.38 (1.23–1.55)	33 (20–47)	1.22 (1.09–1.38)	21 (8–35)
Pulmonary condition or allergy	755/29,232	1.45 (1.32–1.59)	80 (56–104)	1.34 (1.21–1.47)	64 (40–88)
Gastrointestinal condition	326/45,452	1.34 (1.16–1.55)	20 (9–32)	1.29 (1.12–1.49)	18 (7–29)
Urogenital condition	195/47,253	1.40 (1.17–1.67)	17 (7–28)	1.34 (1.12–1.61)	15 (5–26)
Musculoskeletal condition	932/28,704	1.30 (1.20–1.41)	81 (53–109)	1.26 (1.16–1.37)	71 (43–99)
Hematologic condition	201/47,758	1.69 (1.41–2.02)	21 (12–31)	1.49 (1.24–1.79)	17 (8–26)
Neurologic condition	622/36,506	1.31 (1.18–1.44)	45 (26–64)	1.26 (1.14–1.40)	39 (20–59)
Cancer	348/46,682	1.14 (1.00–1.30)	11 (-1–23)	1.14 (1.00–1.30)	11 (-1–23)
**Social isolation**					
Mental disorder	126/18,495	3.92 (3.07–4.99)	111 (76–146)	2.90 (2.28–3.69)	82 (53–111)
All-cause dementia	136/22,345	1.30 (1.04–1.62)	5 (0–10)	1.25 (1.00–1.56)	4 (-0–9)
Circulatory condition	420/7,374	1.24 (1.09–1.40)	52 (18–85)	1.10 (0.97–1.25)	22 (-9–54)
Endocrine condition	267/17,226	1.33 (1.15–1.54)	29 (12–46)	1.14 (0.98–1.32)	13 (-3–29)
Pulmonary condition or allergy	396/13,876	1.46 (1.29–1.66)	83 (51–116)	1.31 (1.15–1.48)	59 (28–90)
Gastrointestinal condition	237/19,772	1.04 (0.89–1.22)	3 (-8–13)	0.97 (0.82–1.14)	-2 (-12–8)
Urogenital condition	259/19,977	1.23 (1.05–1.44)	10 (2–18)	1.17 (1.00–1.37)	8 (-1–16)
Musculoskeletal condition	521/9,798	1.10 (0.98–1.22)	26 (-6–58)	1.01 (0.91–1.13)	4 (-27–35)
Hematologic condition	225/21,443	1.53 (1.29–1.83)	17 (9–25)	1.35 (1.14–1.61)	12 (4–20)
Neurologic condition	378/13,786	0.91 (0.80–1.04)	-13 (-31–4)	0.87 (0.77–0.99)	-20 (-37–-2)
Cancer	374/20,034	1.13 (1.00–1.28)	10 (-1–21)	1.10 (0.97–1.25)	8 (-3–19)
**Low social support**					
Mental disorder	711/99,402	2.52 (2.27–2.80)	50 (43–58)	2.24 (2.01–2.50)	46 (39–54)
All-cause dementia	227/113,990	1.18 (1.00–1.41)	3 (-0–6)	1.15 (0.97–1.37)	3 (-1–6)
Circulatory condition	2,050/66,549	1.19 (1.13–1.27)	42 (27–56)	1.14 (1.07–1.21)	31 (16–45)
Endocrine condition	1,083/96,656	1.22 (1.13–1.32)	19 (11–27)	1.11 (1.03–1.20)	11 (2–19)
Pulmonary condition or allergy	1,560/72,470	1.19 (1.11–1.27)	34 (20–48)	1.12 (1.04–1.19)	22 (8–36)
Gastrointestinal condition	853/105,554	1.13 (1.04–1.24)	8 (2–14)	1.10 (1.01–1.21)	6 (0–13)
Urogenital condition	605/108,178	1.13 (1.02–1.26)	6 (1–11)	1.10 (0.99–1.22)	4 (-1–10)
Musculoskeletal condition	2,309/68,244	1.13 (1.07–1.20)	36 (20–52)	1.11 (1.05–1.17)	29 (13–46)
Hematologic condition	430/111,380	1.25 (1.10–1.41)	8 (3–12)	1.14 (1.00–1.29)	5 (-0–10)
Neurologic condition	1,577/85,440	1.16 (1.09–1.24)	23 (12–34)	1.13 (1.06–1.21)	19 (8–30)
Cancer	980/106,913	1.01 (0.93–1.09)	0 (-6–7)	1.00 (0.93–1.09)	0 (-6–7)
**Composite measure**					
Mental disorder	966/125,993	2.94 (2.66–3.24)	58 (51–64)	2.63 (2.38–2.91)	54 (47–61)
All-cause dementia	355/147,538	1.35 (1.16–1.57)	6 (3–9)	1.31 (1.13–1.52)	5 (2–8)
Circulatory condition	2,592/85,084	1.24 (1.17–1.30)	50 (37–63)	1.17 (1.11–1.23)	38 (24–51)
Endocrine condition	1,425/124,413	1.27 (1.19–1.37)	23 (16–30)	1.15 (1.07–1.24)	14 (7–22)
Pulmonary condition or allergy	2,092/93,324	1.26 (1.19–1.34)	45 (33–58)	1.18 (1.11–1.25)	33 (20–46)
Gastrointestinal condition	1,080/136,426	1.12 (1.03–1.21)	7 (2–12)	1.08 (1.00–1.17)	5 (-0–10)
Urogenital condition	813/139,695	1.17 (1.06–1.28)	7 (3–12)	1.13 (1.03–1.25)	6 (1–10)
Musculoskeletal condition	3,013/87,007	1.15 (1.10–1.21)	40 (25–54)	1.12 (1.06–1.17)	32 (17–46)
Hematologic condition	631/144,196	1.32 (1.18–1.48)	10 (6–14)	1.19 (1.07–1.34)	7 (2–11)
Neurologic condition	2,057/109,230	1.14 (1.08–1.21)	21 (11–30)	1.11 (1.05–1.18)	16 (7–26)
Cancer	1,316/138,232	1.04 (0.97–1.12)	3 (-3–9)	1.04 (0.96–1.11)	3 (-3–9)

CI: Confidence interval; IRR: Incidence rate ratio. IRD: Incidence rate difference. Missing data was imputed using multiple imputation by chained equations. The number of events and person-years at risk are unweighted, whereas the results are weighted based on register data to represent the population of the included regions in 2013 and 2017. The estimates are adjusted for age, sex, and year of survey participation in Model 1, and further adjusted for country of birth, educational level, income, and wealth in Model 2. IRDs are calculated using marginal standardization to individuals with versus without at least one of loneliness, social isolation, and low social support.

Table S6. Social disconnectedness and sex-stratified rates of subsequent medical conditions in 11 broad categories in four regions of Denmark, 2013-2021

	Women			Men		
	Events/person-years at risk among exposed	IRR (95% CI)	IRD per 10,000 person-years (95% CI)	Events/person-years at risk among exposed	IRR (95% CI)	IRD per 10,000 person-years (95% CI)
**Loneliness**						
Mental disorder	324/21,086	3.02 (2.59–3.53)	89 (70–107)	175/15,018	3.29 (2.67–4.06)	76 (56–96)
All-cause dementia	58/29,289	1.67 (1.19–2.33)	13 (2–23)	39/19,660	1.88 (1.27–2.79)	13 (3–24)
Circulatory condition	452/18,855	1.44 (1.28–1.62)	90 (57–124)	334/12,292	1.19 (1.03–1.37)	47 (6–88)
Endocrine condition	294/24,320	1.20 (1.04–1.40)	22 (3–41)	190/17,158	1.24 (1.03–1.49)	20 (1–40)
Pulmonary condition or allergy	461/16,432	1.38 (1.22–1.56)	78 (45–111)	293/12,800	1.27 (1.09–1.48)	48 (14–82)
Gastrointestinal condition	174/27,190	1.22 (1.00–1.48)	13 (-1–28)	151/18,263	1.38 (1.12–1.71)	24 (6–42)
Urogenital condition	34/29,331	1.23 (0.79–1.90)	3 (-4–10)	161/17,922	1.35 (1.11–1.65)	27 (7–47)
Musculoskeletal condition	573/16,094	1.30 (1.17–1.45)	95 (52–138)	359/12,610	1.17 (1.03–1.34)	43 (5–81)
Hematologic condition	120/28,417	1.56 (1.23–1.98)	20 (7–32)	81/19,342	1.42 (1.06–1.90)	14 (1–27)
Neurologic condition	387/21,157	1.27 (1.12–1.45)	44 (18–71)	235/15,350	1.23 (1.05–1.44)	33 (5–60)
Cancer	170/27,833	1.06 (0.87–1.29)	4 (-11–19)	177/18,849	1.23 (1.03–1.48)	20 (1–39)
**Social isolation**						
Mental disorder	60/8,548	2.36 (1.65–3.39)	70 (27–112)	66/9,947	3.29 (2.38–4.55)	83 (46–121)
All-cause dementia	78/10,248	1.36 (0.99–1.86)	7 (-1–15)	58/12,097	1.12 (0.81–1.56)	2 (-4–8)
Circulatory condition	185/3,139	1.19 (0.98–1.43)	39 (-8–86)	234/4,234	1.04 (0.88–1.24)	11 (-34–56)
Endocrine condition	109/7,631	1.08 (0.86–1.37)	9 (-19–37)	159/9,595	1.14 (0.94–1.39)	12 (-7–31)
Pulmonary condition or allergy	166/5,782	1.29 (1.06–1.56)	60 (8–111)	230/8,095	1.32 (1.11–1.55)	57 (18–95)
Gastrointestinal condition	105/9,155	1.01 (0.79–1.30)	1 (-15–16)	132/10,616	0.93 (0.75–1.16)	-4 (-17–9)
Urogenital condition	60/10,279	1.66 (1.18–2.35)	8 (2–15)	199/9,698	1.08 (0.90–1.29)	6 (-9–20)
Musculoskeletal condition	221/3,566	1.06 (0.90–1.25)	19 (-36–75)	300/6,231	0.97 (0.84–1.12)	-7 (-42–29)
Hematologic condition	105/9,797	1.48 (1.13–1.92)	17 (4–30)	121/11,646	1.23 (0.97–1.56)	8 (-2–17)
Neurologic condition	166/5,833	0.80 (0.66–0.97)	-33 (-60–-7)	212/7,953	0.92 (0.78–1.09)	-12 (-34–11)
Cancer	147/9,222	1.14 (0.93–1.39)	10 (-6–26)	226/10,812	1.07 (0.91–1.25)	6 (-9–20)
**Low social support**						
Mental disorder	411/47,099	2.29 (1.98–2.65)	57 (44–69)	299/52,303	2.19 (1.86–2.59)	38 (28–48)
All-cause dementia	111/55,714	1.12 (0.87–1.44)	2 (-3–8)	116/58,276	1.18 (0.93–1.50)	3 (-1–7)
Circulatory condition	936/32,348	1.20 (1.10–1.31)	41 (20–61)	1,114/34,201	1.09 (1.01–1.18)	23 (2–44)
Endocrine condition	548/45,714	1.07 (0.96–1.20)	8 (-5–21)	535/50,942	1.15 (1.03–1.29)	13 (2–23)
Pulmonary condition or allergy	785/32,742	1.20 (1.09–1.32)	42 (19–64)	775/39,728	1.04 (0.94–1.14)	6 (-12–25)
Gastrointestinal condition	408/51,259	1.14 (1.00–1.29)	8 (-0–17)	445/54,296	1.08 (0.95–1.22)	5 (-3–13)
Urogenital condition	71/55,616	1.20 (0.89–1.63)	3 (-2–7)	534/52,561	1.09 (0.97–1.22)	7 (-2–16)
Musculoskeletal condition	1,167/30,311	1.17 (1.08–1.26)	52 (25–79)	1,142/37,933	1.05 (0.97–1.13)	13 (-7–32)
Hematologic condition	205/53,992	1.21 (1.01–1.46)	7 (-0–15)	225/57,388	1.06 (0.89–1.27)	2 (-4–8)
Neurologic condition	775/39,410	1.15 (1.05–1.26)	25 (8–42)	801/46,031	1.10 (1.01–1.21)	15 (0–29)
Cancer	404/51,906	0.98 (0.86–1.12)	-1 (-11–8)	577/55,007	1.02 (0.92–1.13)	2 (-8–11)
**Composite measure**						
Mental disorder	571/62,287	2.56 (2.25–2.92)	61 (51–72)	395/63,706	2.72 (2.33–3.18)	48 (39–57)
All-cause dementia	179/75,345	1.25 (1.01–1.55)	5 (-0–10)	176/72,193	1.37 (1.12–1.69)	5 (2–9)
Circulatory condition	1,235/43,779	1.24 (1.14–1.34)	48 (29–66)	1,357/41,305	1.11 (1.03–1.20)	28 (8–47)
Endocrine condition	745/61,701	1.13 (1.02–1.24)	13 (2–25)	680/62,713	1.17 (1.05–1.30)	14 (4–24)
Pulmonary condition or allergy	1,072/44,278	1.22 (1.12–1.33)	45 (25–65)	1,020/49,046	1.13 (1.03–1.23)	22 (5–39)
Gastrointestinal condition	524/69,529	1.09 (0.97–1.22)	5 (-2–13)	556/66,897	1.08 (0.96–1.21)	5 (-3–13)
Urogenital condition	123/75,282	1.36 (1.06–1.74)	4 (1–8)	690/64,413	1.09 (0.99–1.21)	7 (-1–15)
Musculoskeletal condition	1,569/40,631	1.17 (1.10–1.25)	53 (30–77)	1,444/46,376	1.06 (0.98–1.13)	14 (-4–32)
Hematologic condition	311/73,155	1.27 (1.08–1.49)	9 (3–16)	320/71,041	1.11 (0.96–1.30)	4 (-2–9)
Neurologic condition	1,050/53,115	1.12 (1.03–1.22)	20 (5–35)	1,007/56,115	1.09 (1.00–1.18)	12 (-1–25)
Cancer	556/70,380	1.02 (0.91–1.14)	1 (-7–10)	760/67,851	1.04 (0.95–1.15)	4 (-5–12)

CI: Confidence interval. IRR: Incidence rate ratio. IRD: Incidence rate difference. Missing data was imputed using multiple imputation by chained equations. The number of events and person-years at risk are unweighted, whereas the results are weighted based on register data to represent the population of the included regions in 2013 and 2017. IRDs are calculated using marginal standardization to individuals with versus without at least one of loneliness, social isolation, and low social support. All estimates are adjusted for age, year of survey participation, country of birth, educational level, income, and wealth (Model 2).

Table S7. Social disconnectedness and age-stratified rates of subsequent medical conditions in 11 broad categories in four regions of Denmark, 2013-2021

	**16-65 years**			**>65 years**		
	Events/person-years at risk among exposed	IRR (95% CI)	IRD per 10,000 person-years (95% CI)	Events/person-years at risk among exposed	IRR (95% CI)	IRD per 10,000 person-years (95% CI)
**Loneliness**						
Mental disorder	452/28,844	3.08 (2.70–3.51)	93 (77–109)	46/7,260	3.90 (2.63–5.78)	49 (25–73)
All-cause dementia*	–	–	–	89/7,830	1.83 (1.40–2.39)	61 (26–96)
Circulatory condition	664/29,857	1.35 (1.22–1.49)	63 (39–87)	121/1,291	1.11 (0.88–1.39)	90 (-126–307)
Endocrine condition	386/35,697	1.26 (1.10–1.44)	21 (8–34)	98/5,781	1.08 (0.84–1.38)	13 (-30–55)
Pulmonary condition or allergy	613/25,050	1.32 (1.19–1.47)	57 (33–81)	141/4,183	1.35 (1.08–1.67)	87 (15–159)
Gastrointestinal condition	227/38,641	1.42 (1.19–1.69)	18 (7–28)	99/6,811	1.01 (0.78–1.31)	1 (-36–39)
Urogenital condition	93/40,316	1.32 (1.01–1.72)	7 (-0–14)	102/6,937	1.33 (1.03–1.71)	47 (-0–95)
Musculoskeletal condition	727/26,437	1.23 (1.12–1.35)	49 (24–73)	205/2,267	1.31 (1.09–1.58)	214 (52–376)
Hematologic condition	79/40,126	1.31 (0.98–1.76)	5 (-1–10)	122/7,633	1.59 (1.26–2.02)	64 (24–103)
Neurologic condition	458/32,825	1.43 (1.27–1.61)	41 (25–57)	164/3,682	0.91 (0.75–1.12)	-39 (-124–46)
Cancer	184/39,895	1.16 (0.96–1.40)	7 (-2–15)	164/6,787	1.10 (0.91–1.32)	23 (-26–72)
**Social isolation**						
Mental disorder	84/5,679	3.58 (2.69–4.76)	133 (82–183)	43/12,816	1.83 (1.22–2.74)	15 (2–27)
All-cause dementia*	–	–	–	131/13,406	1.26 (1.01–1.58)	20 (-1–40)
Circulatory condition	193/4,852	1.22 (1.03–1.46)	41 (2–81)	226/2,522	0.96 (0.81–1.14)	-35 (-179–109)
Endocrine condition	102/7,231	1.13 (0.89–1.43)	11 (-12–33)	165/9,995	1.12 (0.93–1.35)	19 (-14–52)
Pulmonary condition or allergy	152/5,501	1.44 (1.19–1.75)	79 (29–128)	244/8,375	1.18 (1.01–1.39)	46 (-1–94)
Gastrointestinal condition	81/8,037	1.19 (0.90–1.57)	8 (-6–22)	156/11,735	0.87 (0.71–1.07)	-18 (-44–8)
Urogenital condition	49/8,531	1.20 (0.83–1.73)	4 (-5–14)	210/11,446	1.15 (0.96–1.38)	22 (-8–51)
Musculoskeletal condition	157/4,743	1.00 (0.82–1.23)	1 (-44–46)	364/5,054	1.02 (0.90–1.16)	14 (-74–102)
Hematologic condition	40/8,604	2.05 (1.38–3.04)	16 (4–27)	185/12,839	1.22 (1.00–1.47)	23 (-1–48)
Neurologic condition	111/6,673	1.19 (0.95–1.49)	19 (-8–45)	267/7,114	0.77 (0.67–0.90)	-106 (-161–-52)
Cancer	73/8,478	1.13 (0.85–1.50)	5 (-8–18)	301/11,556	1.09 (0.95–1.25)	21 (-15–58)
**Low social support**						
Mental disorder	617/72,906	2.21 (1.97–2.49)	54 (44–64)	94/26,496	2.43 (1.83–3.23)	23 (13–33)
All-cause dementia*	–	–	–	204/27,420	1.12 (0.93–1.34)	9 (-6–24)
Circulatory condition	1,562/60,782	1.16 (1.08–1.24)	29 (15–43)	488/5,767	1.03 (0.92–1.16)	26 (-76–127)
Endocrine condition	752/75,853	1.10 (1.00–1.22)	8 (-0–17)	331/20,803	1.13 (0.98–1.29)	20 (-4–44)
Pulmonary condition or allergy	1,106/55,892	1.10 (1.01–1.19)	17 (2–33)	454/16,579	1.15 (1.02–1.30)	37 (3–71)
Gastrointestinal condition	479/81,538	1.13 (1.00–1.28)	6 (-0–12)	374/24,016	1.05 (0.92–1.20)	7 (-12–26)
Urogenital condition	235/84,572	1.09 (0.92–1.30)	2 (-2–6)	369/23,606	1.10 (0.96–1.26)	14 (-7–35)
Musculoskeletal condition	1,602/57,498	1.14 (1.06–1.21)	29 (14–45)	708/10,746	1.01 (0.92–1.11)	8 (-58–75)
Hematologic condition	135/84,914	0.98 (0.77–1.24)	-0 (-4–3)	294/26,466	1.21 (1.04–1.40)	22 (3–42)
Neurologic condition	915/70,596	1.22 (1.12–1.33)	21 (12–31)	662/14,845	1.01 (0.91–1.11)	3 (-43–48)
Cancer	409/83,437	0.95 (0.83–1.08)	-2 (-7–3)	571/23,476	1.05 (0.94–1.16)	11 (-15–38)
**Composite measure**						
Mental disorder	843/88,711	2.66 (2.38–2.96)	65 (56–74)	122/37,283	2.40 (1.81–3.17)	21 (13–29)
All-cause dementia	28/108,969	1.25 (0.72–2.18)	1 (-1–2)	327/38,569	1.30 (1.11–1.52)	21 (8–35)
Circulatory condition	1,922/77,171	1.22 (1.15–1.30)	39 (26–52)	669/7,913	1.00 (0.90–1.11)	2 (-87–90)
Endocrine condition	959/95,268	1.17 (1.07–1.28)	13 (5–21)	466/29,145	1.11 (0.99–1.26)	18 (-3–39)
Pulmonary condition or allergy	1,435/69,707	1.17 (1.09–1.26)	29 (15–44)	657/23,617	1.18 (1.06–1.31)	43 (14–72)
Gastrointestinal condition	598/102,556	1.19 (1.06–1.34)	8 (3–13)	482/33,869	0.94 (0.84–1.07)	-8 (-25–9)
Urogenital condition	289/106,532	1.14 (0.96–1.34)	3 (-1–7)	525/33,163	1.12 (0.99–1.26)	17 (-2–36)
Musculoskeletal condition	1,997/72,168	1.16 (1.09–1.23)	33 (19–48)	1,016/14,839	1.01 (0.93–1.10)	6 (-52–64)
Hematologic condition	184/106,851	1.09 (0.88–1.34)	1 (-2–5)	447/37,345	1.23 (1.08–1.40)	24 (8–40)
Neurologic condition	1,165/88,623	1.28 (1.18–1.38)	26 (17–35)	892/20,607	0.93 (0.85–1.01)	-34 (-72–5)
Cancer	505/105,155	0.98 (0.87–1.11)	-1 (-6–4)	811/33,077	1.07 (0.97–1.17)	16 (-7–39)

CI: Confidence interval. IRR: Incidence rate ratio. IRD: Incidence rate difference. Missing data was imputed using multiple imputation by chained equations. The number of events and person-years at risk are unweighted, whereas the results are weighted based on register data to represent the population of the included regions in 2013 and 2017. IRDs are calculated using marginal standardization to individuals with versus without at least one of loneliness, social isolation, and low social support. All estimates are adjusted for sex, age, year of survey participation, country of birth, educational level, income, and wealth (Model 2).
* Results for each separate aspect of social disconnectedness are not provided among individuals aged 16-65 years due to few cases of all-cause dementia in this age group.

Table S8. Interaction between social disconnectedness and pre-existing mental disorders on rates of subsequent medical conditions in 10 broad categories in four regions of Denmark, 2013-2021

	**Without a mental disorder**		**With a mental disorder**		**Expected with a mental disorder**	
	Events/person-years at risk	IRR (95% CI)	Events/person-years at risk	IRR (95% CI)	IRR (95% CI)	RERI (95% CI)
**All-cause dementia**						
Reference	1,031/699,601	1 (ref.)	52/34,176	2.47 (1.77–3.44)		
Composite measure	330/127,758	1.33 (1.14–1.56)	25/19,780	2.07 (1.27–3.35)	2.80 (1.93–3.67)	-0.73 (-2.06–0.59)
**Circulatory condition**						
Reference	11,422/425,244	1 (ref.)	521/22,523	1.35 (1.21–1.51)		
Composite measure	2,229/72,980	1.16 (1.10–1.23)	362/12,104	1.67 (1.47–1.90)	1.52 (1.35–1.68)	0.16 (-0.11–0.42)
**Endocrine condition**						
Reference	5,746/609,564	1 (ref.)	340/28,688	1.51 (1.32–1.72)		
Composite measure	1,219/107,931	1.14 (1.06–1.23)	206/16,482	1.47 (1.25–1.75)	1.65 (1.43–1.88)	-0.18 (-0.50–0.14)
**Pulmonary condition or allergy**						
Reference	8,229/462,491	1 (ref.)	445/19,554	1.35 (1.20–1.51)		
Composite measure	1,775/82,599	1.14 (1.07–1.22)	317/10,725	1.69 (1.48–1.93)	1.49 (1.31–1.67)	0.20 (-0.08–0.48)
**Gastrointestinal condition**						
Reference	4,646/655,590	1 (ref.)	211/31,703	1.36 (1.15–1.62)		
Composite measure	938/118,448	1.06 (0.98–1.16)	143/17,978	1.56 (1.27–1.92)	1.42 (1.17–1.68)	0.14 (-0.27–0.54)
**Urogenital condition**						
Reference	3,140/665,616	1 (ref.)	81/33,318	0.98 (0.75–1.28)		
Composite measure	749/120,358	1.29 (1.17–1.42)	64/19,337	1.10 (0.81–1.49)	1.28 (0.98–1.57)	-0.18 (-0.62–0.26)
**Musculoskeletal condition**						
Reference	14,107/455,874	1 (ref.)	699/20,397	1.49 (1.36–1.63)		
Composite measure	2,634/76,498	1.08 (1.02–1.14)	378/10,509	1.53 (1.35–1.73)	1.56 (1.41–1.72)	-0.04 (-0.28–0.20)
**Hematologic condition**						
Reference	2,056/687,934	1 (ref.)	113/33,384	1.93 (1.52–2.45)		
Composite measure	563/125,029	1.21 (1.08–1.36)	69/19,167	1.89 (1.40–2.54)	2.15 (1.65–2.64)	-0.26 (-0.99–0.47)
**Neurologic condition**						
Reference	9,407/543,691	1 (ref.)	414/25,721	1.34 (1.19–1.51)		
Composite measure	1,804/94,775	1.07 (1.01–1.14)	253/14,454	1.53 (1.32–1.78)	1.41 (1.24–1.59)	0.12 (-0.16–0.40)
**Cancer**						
Reference	6,011/648,403	1 (ref.)	196/32,712	1.08 (0.90–1.28)		
Composite measure	1,196/119,222	1.05 (0.98–1.14)	120/19,010	1.15 (0.92–1.43)	1.13 (0.92–1.34)	0.02 (-0.30–0.34)

CI: Confidence interval. IRR: Incidence rate ratio. RERI: Relative Excess Risk due to Interaction. Missing data was imputed using multiple imputation by chained equations. The number of events and person-years at risk are unweighted, whereas the results are weighted based on register data to represent the population of the included regions in 2013 and 2017. The estimates of expected with a mental disorder are calculated based on additive interaction between mental disorders and the composite measure (at least one of loneliness, social isolation, and low social support). All estimates are adjusted for age, sex, year of survey participation, country of birth, educational level, income, and wealth (Model 2).

References

1. OECD/Eurostat/UNESCO Institute for Statistics. ISCED 2011 operational manual: guidelines for classifying national education programmes and related qualifications [Internet]. Paris: OECD Publishing; 2015. Available from: https://www.oecd-ilibrary.org/education/isced-2011-operational-manual_9789264228368-en

2. OECD. Framework for integrated analysis. In: OECD Framework for Statistics on the Distribution of Household Income, Consumption and Wealth [Internet]. Paris: OECD Publishing; 2013 [cited 2023 Jun 27]. p. 171–92. Available from: https://www.oecd-ilibrary.org/economics/oecd-framework-for-statistics-on-the-distribution-of-household-income-consumption-and-wealth/framework-for-integrated-analysis_9789264194830-11-en

3. Statistics Denmark. TIMES variabel - FAMDISPONIBEL_13 [Internet]. [cited 2023 Jun 30]. Available from: https://www.dst.dk/da/Statistik/dokumentation/Times/familieindkomst/famdisponibel-13

4. Statistics Denmark. TIMES variabel - FAMFORMREST_NY05 [Internet]. [cited 2023 Jun 30]. Available from: https://www.dst.dk/da/Statistik/dokumentation/Times/familieindkomst/famformrest-ny05

5. Bartlett JW, Harel O, Carpenter JR. Asymptotically unbiased estimation of exposure odds ratios in complete records logistic regression. Am J Epidemiol. 2015 Oct 15;182(8):730–6.

6. Hughes RA, Heron J, Sterne JAC, Tilling K. Accounting for missing data in statistical analyses: multiple imputation is not always the answer. Int J Epidemiol. 2019 Aug 1;48(4):1294–304.

7. Morris TP, White IR, Royston P. Tuning multiple imputation by predictive mean matching and local residual draws. BMC Med Res Methodol. 2014 Jun 5;14:75.

8. Wulff JN, Ejlskov L. Multiple imputation by chained equations in praxis: guidelines and review. Electron J Bus. 2017;15(1).

9. White IR, Royston P, Wood AM. Multiple imputation using chained equations: issues and guidance for practice. Stat Med. 2011 Feb 20;30(4):377–99.

10. Lash TL, VanderWeele TJ, Haneuse S, Rothman KJ. Modern epidemiology. Fourth edition. Philadelphia: Wolters Kluwer; 2021. 1250 p.

11. Kessing LV, Ziersen SC, Caspi A, Moffitt TE, Andersen PK. Lifetime incidence of treated mental health disorders and psychotropic drug prescriptions and associated socioeconomic functioning. JAMA Psychiatry [Internet]. 2023 Jul 12; Available from: https://jamanetwork.com/journals/jamapsychiatry/fullarticle/2806888

12. Andersen JS, Olivarius NDF, Krasnik A. The Danish National Health Service Register. Scand J Public Health. 2011 Jul;39(7 Suppl):34–7.

13. Gariépy G, Honkaniemi H, Quesnel-Vallée A. Social support and protection from depression: systematic review of current findings in Western countries. Br J Psychiatry. 2016 Oct;209(4):284–93.

14. Kuiper JS, Zuidersma M, Oude Voshaar RC, Zuidema SU, van den Heuvel ER, Stolk RP, et al. Social relationships and risk of dementia: A systematic review and meta-analysis of longitudinal cohort studies. Ageing Research Reviews. 2015 Jul 1;22:39–57.

15. Lara E, Martín-María N, De la Torre-Luque A, Koyanagi A, Vancampfort D, Izquierdo A, et al. Does loneliness contribute to mild cognitive impairment and dementia? A systematic review and meta-analysis of longitudinal studies. Ageing Research Reviews. 2019 Jul 1;52:7–16.

16. Mann F, Wang J, Pearce E, Ma R, Schlief M, Lloyd-Evans B, et al. Loneliness and the onset of new mental health problems in the general population. Soc Psychiatry Psychiatr Epidemiol. 2022 Nov;57(11):2161–78.

17. Mansfield M, Thacker M, Sandford F. Psychosocial Risk Factors and the Association With Carpal Tunnel Syndrome: A Systematic Review. Hand (N Y). 2018 Sep;13(5):501–8.

18. Penninkilampi R, Casey AN, Singh MF, Brodaty H. The Association between Social Engagement, Loneliness, and Risk of Dementia: A Systematic Review and Meta-Analysis. J Alzheimers Dis. 2018;66(4):1619–33.

19. Santini ZI, Koyanagi A, Tyrovolas S, Mason C, Haro JM. The association between social relationships and depression: a systematic review. J Affect Disord. 2015 Apr 1;175:53–65.

20. Valtorta NK, Kanaan M, Gilbody S, Ronzi S, Hanratty B. Loneliness and social isolation as risk factors for coronary heart disease and stroke: systematic review and meta-analysis of longitudinal observational studies. Heart. 2016 Jul 1;102(13):1009–16.

21. Wang S, Molassiotis A, Guo C, Leung ISH, Leung AYM. Association between social integration and risk of dementia: A systematic review and meta-analysis of longitudinal studies. J Am Geriatr Soc. 2023;71(2):632–45.

22. Yang J, Huang J, Yang X, Li S, Wu X, Ma X. The association of living alone and social isolation with sarcopenia: A systematic review and meta-analysis. Ageing Res Rev. 2023 Nov;91:102043.
